# Dual‐Stimulus Programmed Multiphase Separation and Organization in Coacervate Droplets

**DOI:** 10.1002/anie.202512266

**Published:** 2025-09-07

**Authors:** Yang Zhou, Brigitte Voit, Dietmar Appelhans

**Affiliations:** ^1^ Division Macromolecular Chemistry Leibniz Institute of Polymer Research Dresden Hohe Strasse 6 Dresden 01069 Germany; ^2^ Organic Chemistry of Polymers TUD Dresden University of Technology Dresden 01062 Germany

**Keywords:** Coacervate protocells, Morphological transformation, Multiphase coacervates, Stimuli‐responsive

## Abstract

Stimuli‐responsive (multiphase) coacervates deserve significant attention as cell‐like entities that can adapt to their environment and undergo morphological reconfiguration. In this study, a tandem‐triggered transition system is presented that enables the transformation of single‐phase coacervates into multiphase structures through the sequential application of two external stimuli: pH and salt concentration. A polyanion containing acid‐labile amide bond is incorporated into the membrane‐less coacervates. Upon exposure to an acidic pH, hydrolysis of the amide bond induces charge reversal from polyanion to polycation, triggering the first transition from single‐phase to nested multiphase coacervates. This transformation alters the spatial redistribution and viscosity of coacervate components and influences sequestration behavior toward various (macro) molecules. Subsequently, the introduction of hypertonic environment as secondary stimulus induces selective dissociation and structural reconfiguration of nested multiphase coacervates into vesicular‐like multiphase coacervates, further altering the coacervate components' fluidity and partitioning properties. Notably, the diverse inherent properties of coacervates among this tandem‐triggered transition enables the variation of spatial organization for enzymatic reactions. Overall, the findings demonstrate a strategy for the sequential control of coacervate structural reconfiguration through dual stimuli, providing a versatile platform for the development of programable and adaptive coacervate‐based protocells.

## Graphical abstract

A dual‐stimulus programmed coacervate transformation system is developed. The sequential application of pH and salt concentration induces transition of membrane‐less coacervates into nested multiphase coacervates and, ultimately, into vesicular‐like multiphase coacervates. This transition endows selective client recruitment and exclusion by resultant coacervates, and facilitates the spatial orchestration of enzymatic reactions.

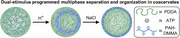

## Introduction

Cells continuously sense and respond to environmental changes to maintain functionality.^[^ ^1^, ^2^, ^3^ ^]^ Inspired by this dynamic adaptability, stimuli‐responsive protocells have garnered significant interest.^[^ ^4^, ^5^ ^]^ As cell‐like entities, protocells exhibit cytomimetic behavior,^[^ ^6^, ^7^ ^]^ with coacervates—formed through liquid–liquid phase separation—being extensively explored.^[^ ^8^, ^9^, ^10^ ^]^ These condensed microscale droplets typically possess a single‐phase structure and demonstrate cell‐like properties such as compartmentalization, substrate enrichment, and confinement of biochemical reactions.^[^ ^11^, ^12^ ^]^ In recent years, stimuli‐responsive single‐phase coacervates are able to respond to external stimuli, such as pH,^[^ ^13^, ^14^, ^15^, ^16^, ^17^ ^]^ salt concentration,^[^ ^18^, ^19^, ^20^ ^]^ light,^[^ ^21^, ^22^, ^23^, ^24^ ^]^ and temperature,^[^ ^25^, ^26^, ^27^ ^]^ enabling dynamic phase separation and achieving precise and flexible control over their properties.^[^ ^4^ ^]^ Additionally, growing insights into membrane‐less multicompartmental organelles in living cells have spurred interest in the application of multiphase coacervates,^[^ ^28^ ^]^ which provide distinct advantages in mimicking complex cell‐like architectures.^[^ ^29^ ^]^ These immiscible coacervate phases enable the spatial isolation of molecules and enzymatic reactions, facilitating biochemical compartmentalization.^[^ ^30^, ^31^, ^32^ ^]^ Following trends in single‐phase coacervates, stimuli‐triggered phase separation has also been introduced in multiphase systems.^[^ ^33^ ^]^ Under the influence of factors, such as temperature,^[^ ^34^ ^]^ pH,^[^ ^35^ ^]^ electric field,^[^ ^36^ ^]^ light,^[^ ^37^ ^]^ or chemical fuel,^[^ ^38^ ^]^ these multiphase coacervates exhibit enhanced dynamic adaptability and responsiveness.

Among various external stimuli, pH, and salt concentration play particularly critical roles in complex coacervation, which relies on electrostatic attraction between oppositely charged polyelectrolytes.^[^ ^39^ ^]^ pH influences the protonation and deprotonation of functional groups, dictating the formation and stability of coacervate droplets with a specific pH window.^[^ ^33^ ^]^ This principle has been leveraged to develop coacervate systems capable of undergoing reversible assembly‐disassembly in single‐phase coacervates^[^ ^40^, ^41^, ^42^, ^43^ ^]^ and morphological transitions in multiphase coacervates.^[^ ^35^ ^]^ Additionally, strategic modifications to polyelectrolytes can induce charge reversal (from positive to negative) in response to pH changes,^[^ ^44^, ^45^ ^]^ offering an alternative approach for controlling the charge state of polyelectrolytes in coacervates and facilitating structural and functional transitions in coacervate‐based protocells. However, despite its potential, charge‐reversal‐driven (multi)phase transitions in coacervate protocells remain unexplored.

Beyond pH, salt concentration serves as another crucial determinant of complex coacervate formation and phase behavior. Increasing salt concentration weakens electrostatic attractions, reducing the entropic driving force of coacervation, and ultimately leads to complete dissolution beyond a critical.^[^ ^39^, ^46^ ^]^ Moreover, the coexistence of distinct phases in complex multiphase coacervates is primarily governed by variations in their critical salt concentrations,^[^ ^47^ ^]^ which can be exploited to generate, stabilize, or selectively dissolve specific phases within multiphase coacervate systems.^[^ ^29^, ^47^, ^48^, ^49^, ^50^ ^]^ Beyond the use of pH and salt concentration in the coexistence of complex multiphase systems, their combination with other (molecular) triggers such as electric field, redox reactions, metabolites, and charge‐reversal‐driven (multi)phase transitions among others may open new (structural) functions in complex coacervate droplets as cell‐like entities.

**Scheme 1:**
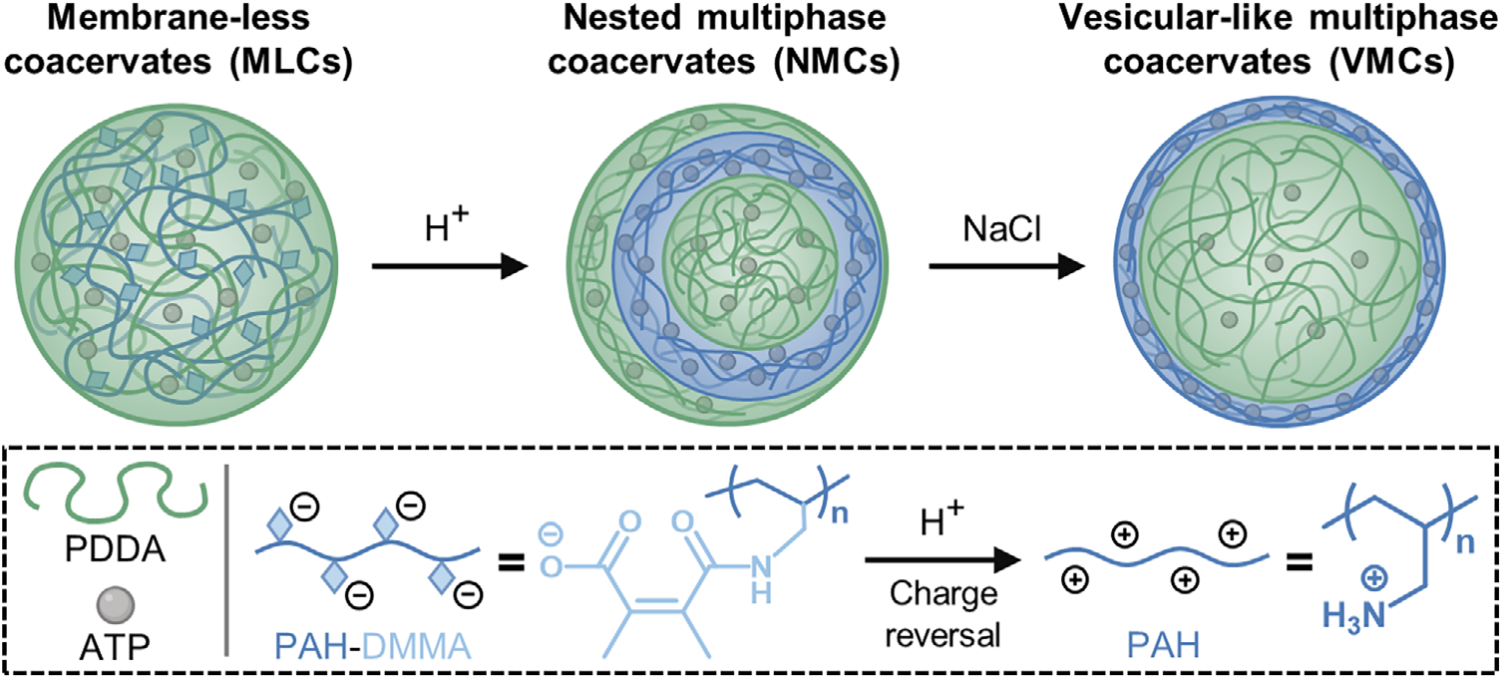
Dual stimuli‐triggered multiphase separation and organization in ternary coacervate droplets. Acid‐cleavable anionic PAH‐DMMA in MLCs transfers into cationic PAH in the presence of H^+^, triggering the initial formation of NMCs composed of immiscible PAH/ATP phase and PDDA/ATP phases. This transformation process presents a charge reversal‐driven multiphase transition. The following addition of NaCl induces the selective dissociation and reconfiguration in the NMCs, resulting in VMCs. Compartments of ATP/PDDA in NMCs and VMCs are considered ATP‐poor and PDDA‐rich areas with sequestration potential, while immiscible PAH/ATP phases in NMCs and VMCs can act as diffusion barrier or as sequestration area for various types of cargo.

**Figure 1:**
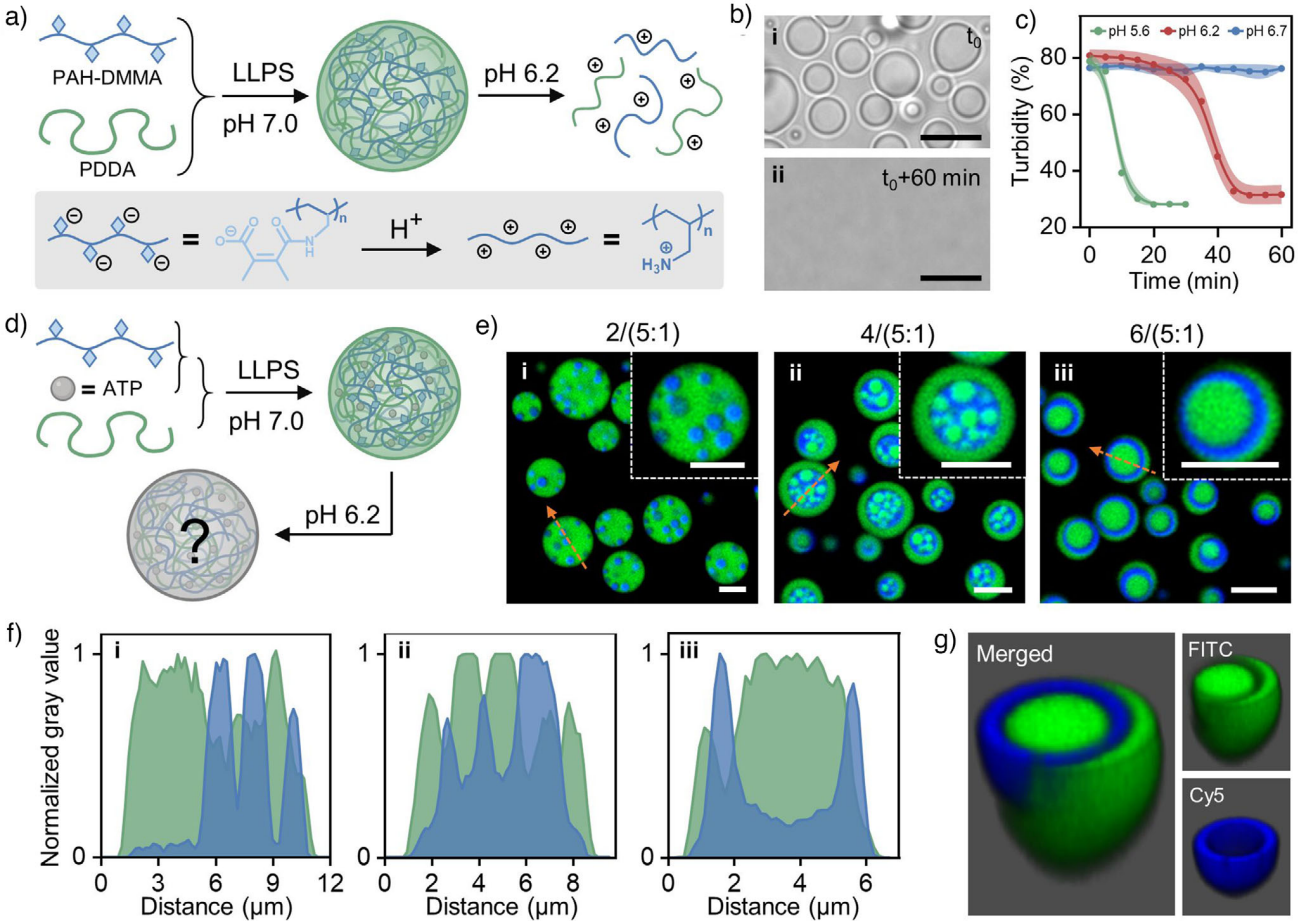
pH‐triggered structural transition in MLCs. a) Schematic illustration for the formation of MLCs from PDDA and PAH‐DMMA and followed by dissociation under acid environment. b) CLSM images (bright field) showing the disassembly of coacervates under pH 6.2 after 60 min. c) Turbidity of PDDA/PAH‐DMMA coacervates over time under pH 6.7, 6.2, and 5.6 (data are mean ± SD, *n* = 3). d) Schematic illustration for the formation of MLCs established by three components (PAH‐DMMA, ATP, and PDDA), followed by structural transitions under acid environment. e) Representative CLSM images of multiphase coacervates generated from MLCs containing three components after ∼5 h at pH 6.2, using *z*/(*x*:*y*) to represent the component weight ratio adopted, where *x*:*y* indicating the weight ratio (ATP/PAH‐DMMA), and *z* indicating the weight ratio (Polyanions/PDDA) (green: fluorescein labeled PDDA (FITC‐PDDA); blue: Cyanine5 labeled PAH (Cy5‐PAH)). f) Normalized gray value across the orange dashed line in e). g) 3D reconstructed image of NMCs generated at a 6/(5:1) ratio (Green: FITC‐PDDA; blue: Cy5‐PAH). Scale bar: 5 µm.

Given the pivotal role of pH and salt concentration in regulating coacervate structure and function, herein, we propose a multiphase transition system in complex coacervate droplets that is sequentially triggered by these two external stimuli (Scheme 1), starting from membrane‐less coacervates (MLCs). To achieve this, poly(allylamine hydrochloride) (PAH) is chemically modified with 2,3‐dimethylmaleic anhydride (DMMA), yielding negatively charged PAH‐DMMA due to the introduction of carboxyl groups. This polyanion is employed as one of the key components to establish ternary complex coacervate droplets (MLCs) at neutral pH. Under mildly acidic conditions, the degradation of β‐carboxylic amide bonds exposes positively charged amino groups on PAH, leading to a charge redistribution among the polyelectrolytes. This pH‐triggered charge‐reversal event initiates the transition from single‐phase to nested multiphase coacervates (NMCs, Scheme 1), enabling tunable architectures (Figure 1), modulating fluidity (Figure 3), and altering sequestration behavior (Figure 4). Following this initial transition, the introduction of a hypertonic environment via NaCl addition serves as a secondary stimulus, triggering selective dissociation and structural reconfiguration within the NMCs, leading to vesicular multiphase coacervates (VMCs, Scheme 1). This process further alters the fluidity of coacervate components and their partitioning properties into polyelectrolyte‐rich and ‐poor areas (Figure 5), facilitating intra‐droplet transfer of biomacromolecules within coacervates (Figure 5). Moreover, the diverse partitioning properties of coacervates in this tandem‐triggered transition enable spatial organization of enzymatic reactions (Figure 6). Overall, this study presents complex architectures in multiphase coacervate systems which are regulated by tandem stimuli (pH and salt concentration), offering a novel strategy for the design of programmable and adaptive coacervate protocells with diverse architectures and functions.

## Results and Discussion

### Phase Transitions of Coacervate Droplets Based on pH‐Triggered Charge‐Reversal Responsivity

As the key component in this system, negatively charged PAH‐DMMA is synthesized as previously reported^[^ ^44^ ^]^ (synthesis and characterization shown in Scheme S1 and Figure S1). To evaluate the charge‐reversal‐induced responsivity of PAH‐DMMA in coacervate droplets, binary coacervates composed of anionic PAH‐DMMA and cationic poly(diallyldimethylammonium chloride) (PDDA, 100–200 kDa) are first prepared at pH 7.0, using a weight ratio of 6:1 (Figure 1a). As shown in Figure 1b(i), confocal laser scanning microscopy (CLSM) image confirms the formation of micron‐sized spherical droplets. Upon the addition of MES buffer solution (10 mM, pH 6.2), the ambient pH decreases to 6.2, triggering the hydrolysis of β‐carboxylic amide bonds in PAH‐DMMA and the generation of PAH with primary amine groups. This charge reversal induces electrostatic repulsion between the two polycations, PAH and PDDA, leading to the dissociation of coacervate droplets after 60 min (Figure 1b(ii)). Since the kinetics of β‐carboxylic amide bond hydrolysis are pH‐dependent, the use of lower pH values results in the acceleration of this charge‐reversal process.^[^ ^51^ ^]^ Therefore, the dissociation behavior of the coacervates is examined at different pH levels (pH 5.6, 6.2, and 6.7). As shown in Figure 1c, the turbidity of the coacervate suspension remains unchanged in 60 min at pH 6.7, indicating minimal production of primary amine groups. In contrast, at pH 6.2, the solution becomes clear after 40 min, suggesting complete dissociation of the coacervate phase. A further decrease in pH to 5.6 leads to the rapid disappearance of coacervates within 20 min, attributed to the faster acid‐driven charge reversal process. These findings strongly support the feasibility of charge‐reversal process to regulate coacervate phase behavior in multicomponent systems. This may enable to generate a more programmable design for dynamic coacervate protocells, such as the release of encapsulated cargos by pH modulation.

Encouraged by these results, we extend our investigation to multiphase coacervates. Adenosine 5′‐triphosphate (ATP) is introduced as the third component alongside PAH‐DMMA and PDDA to establish pH‐treated coacervates with heterogeneously distributed polyelectrolytes. Experimentally, the ternary membrane‐less coacervates (MLCs) are prepared by mixing PDDA with a premixed solution of PAH‐DMMA and ATP at pH 7.0, the subsequent phase transition is triggered by decreasing the pH to 6.2 through the addition of MES buffer solution (10 mM, pH 6.2) (Figure 1d), as previously described (Figure 1a). Given the complexity of this system, which comprises three different polyelectrolytes, we first investigate the potential for structural transitions in coacervate droplets prepared at various weight ratios. To describe the different MLCs compositions, the notation *z*/(*x*:*y*) is defined, where *x*:*y* represents the weight ratio of ATP to PAH‐DMMA, and *z* denotes the weight ratio of polyanions (sum of ATP and PAH‐DMMA) to PDDA (Table S1). Interestingly, MLCs with different component ratios yield multiphase coacervates with distinct morphologies after 5 h of incubation at pH 6.2. As shown in Figures 1e,f and S2, the 2/(5:1) ratio produces stable multiphase coacervates, in which the smaller PAH/ATP phases are preferentially localized within the PDDA/ATP phase (Figure 1e(i), f(i)). The 4/(5:1) ratio produces a more complex hierarchical structure. Numerous smaller PDDA/ATP phases are encapsulated by the PAH/ATP phase, which is entirely surrounded by the PDDA/ATP phase (Figures 1e(ii), f(ii) and S3). Over the subsequent 24 h, these inner PDDA/ATP domains undergo fusion, ultimately forming a single, larger PDDA/ATP phase (Figure S3). Notably, at 6/(5:1), a circular‐like PAH/ATP phase separates the PDDA/ATP phases into distinct internal and external regions (Figure 1e(iii),f(iii)). Due to their well‐defined nested architecture in the reconstructed 3D image (Figures 1g and S4), the nested multiphase coacervates (NMCs) generated at a 6/(5:1) ratio, with structural stability in 24 h (Figure S5), are selected for further investigation.

### pH‐Triggered Transition from Single‐Phase Coacervates to Nested Multiphase Coacervates

**Figure 2:**
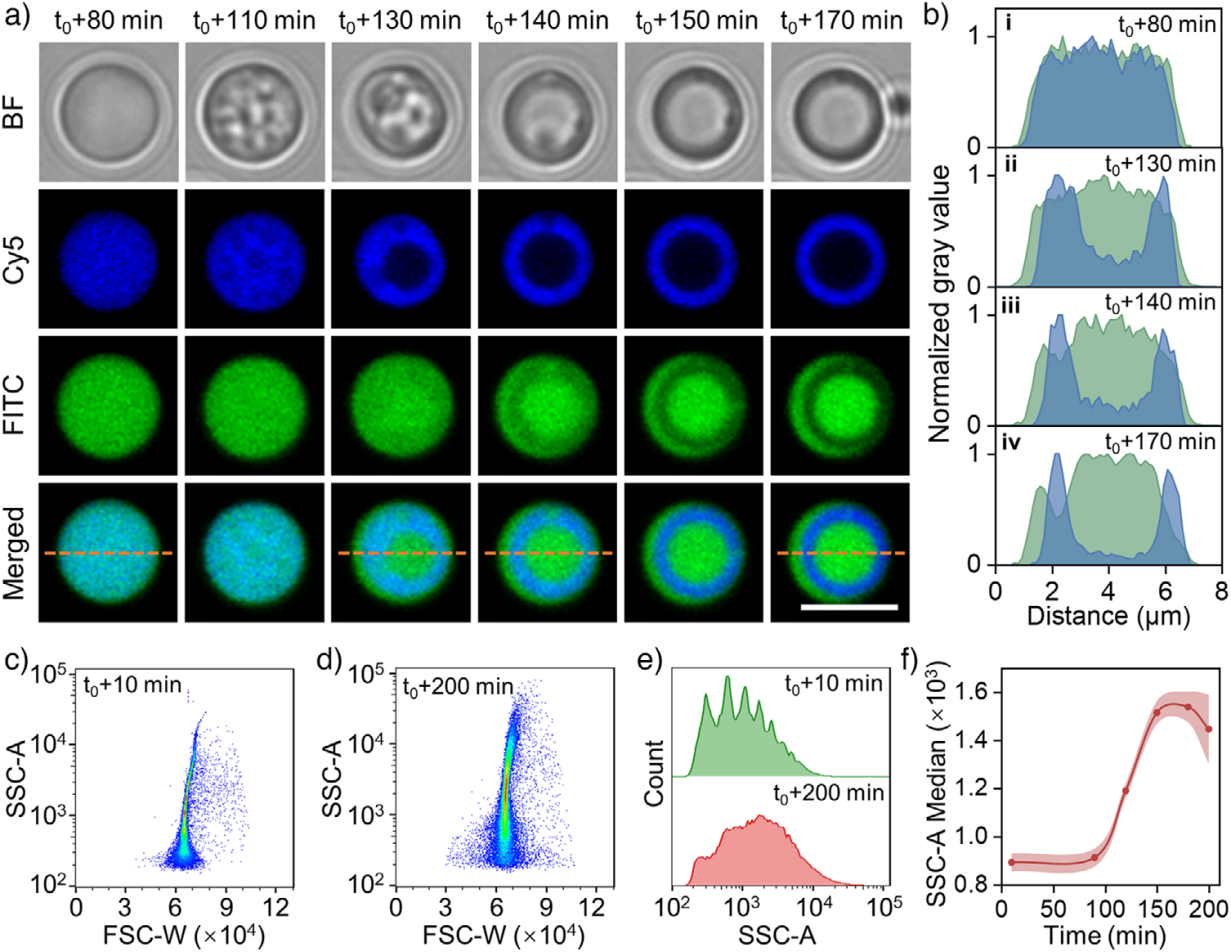
pH‐triggered transition toward NMCs. a) Time series of CLSM images (bright field and fluorescence field) showing the transition from a single MLC to NMC (green: FITC‐PDDA; blue: Cy5‐PAH‐DMMA). b) Normalized gray value across the orange dashed line from the merged channel at different time points in a). c), d) 2D pseudocolor dot plots of FSC‐W versus SSC‐A recorded at c) 10 min and d) 200 min in the transition. e) Corresponding SSC‐A histograms of coacervates population in c) and d), respectively. f) Time‐dependent SSC‐A median change during this transition (data are mean ± SD, *n* = 3). Scale bar: 5 µm.

To better understand the pH‐triggered transition process into NMCs, experimentally, an in situ CLSM study is performed. MLCs with a component ratio of 6/(5:1) are transferred into a chamber slide and monitored under undisturbed conditions at pH 6.2. As shown in Figures 2a and S6, Cy5‐PAH and FITC‐PDDA initially exhibit a homogeneous distribution within the coacervate matrix, and preserving structural uniformity for a duration of up to 80 min under acidic environment (Figure 2b(i)). Over time, small dilute aqueous phases begin to emerge in the Cy5‐PAH channel, gradually increasing in size (110–130 min), while FITC‐PDDA remains homogeneously dispersed (Figure 2a,b(ii)). As the transition progresses, these dilute aqueous phases continue to expand and coalesce, eventually forming a circular structure in the Cy5‐PAH channel. Simultaneously, FITC‐PDDA is progressively excluded from the Cy5‐PAH‐rich region, ultimately segregating into distinct inner and outer phases (140–170 min) (Figure 2a,b(iii,iv)). Notably, the overall size of the coacervate remains unchanged throughout this transition (Figure S6). A similar phase redistribution is observed for ATP, which displays a spatial distribution pattern analogous to that of PAH (Figure S7). This behavior is attributed to the stronger electrostatic attraction between PAH and ATP compared to PDDA and ATP after the acidic degradation of PAH‐DMMA. Consequently, the resulting NMCs consist of two ATP‐poor, PDDA‐rich outer and inner phases, which are separated by a circular PAH‐ and ATP‐enriched phase (Figure S8). Moreover, the NMCs maintain this nested architecture across a broad pH range (pH 4–8). However, when the pH exceeds the p*K*_a_ of PAH (e.g., at pH 9.5 and 11), the nested structure dissipates, leading to a homogeneous distribution of coacervate components (Figure S9). This transition is attributed to the deprotonation of PAH, which disrupts its electrostatic interactions with ATP. In order to provide further characterization for the structural transition, flow cytometry is employed to analyze the granularity and complexity of coacervate droplets as cell‐like entities during phase separation. Two‐dimensional dot plots of side‐scattered light area (SSC‐A) versus forward‐scattered light width (FSC‐W) reveal the coacervate population within the first 10 min in an acidic environment (Figure 2c), with a median SSC value of approximately 890 (Figure 2e,f). As the phase separation process continues, the formation of the nested structure leads to increased structural heterogeneity and results in a higher SSC median (∼1450) after 200 min (Figure 2d–f). This increase in optical granularity reflects an internal reorganization within the coacervate matrix. Notably, due to the inherent polydispersity of coacervate droplets, both FSC and SSC signals exhibit a broad distribution across all time points.

In addition, this multiphase transition process is smoothly initiated in a controlled manner by a pH‐dependent response, which is attributed to the pH‐sensitive hydrolysis of PAH‐DMMA. This phenomenon is consistent with the observed dissociation behavior in binary coacervates (Figure 1a–c). Under mildly acidic conditions (pH 6.7), the ternary coacervates retain a homogeneous structure after 5 h (Figure S10), indicating minimal hydrolysis. In contrast, at a pH of 5.6, the transition to NMCs occurs more rapidly than at pH of 6.2 (Figures 2a and S6), and the complete phase separation is achieved within 1.5 h (Figure S11). These findings suggest that the kinetics of multiphase coacervate formation can be controlled by adjusting the pH of the medium, offering a tunable strategy for designing programmable phase separation in multiphase coacervate systems.

### pH‐Triggered Transition Endows Diverse Fluidity and Sequestration Behavior in Coacervate Droplets as Cell‐Like Entities

**Figure 3:**
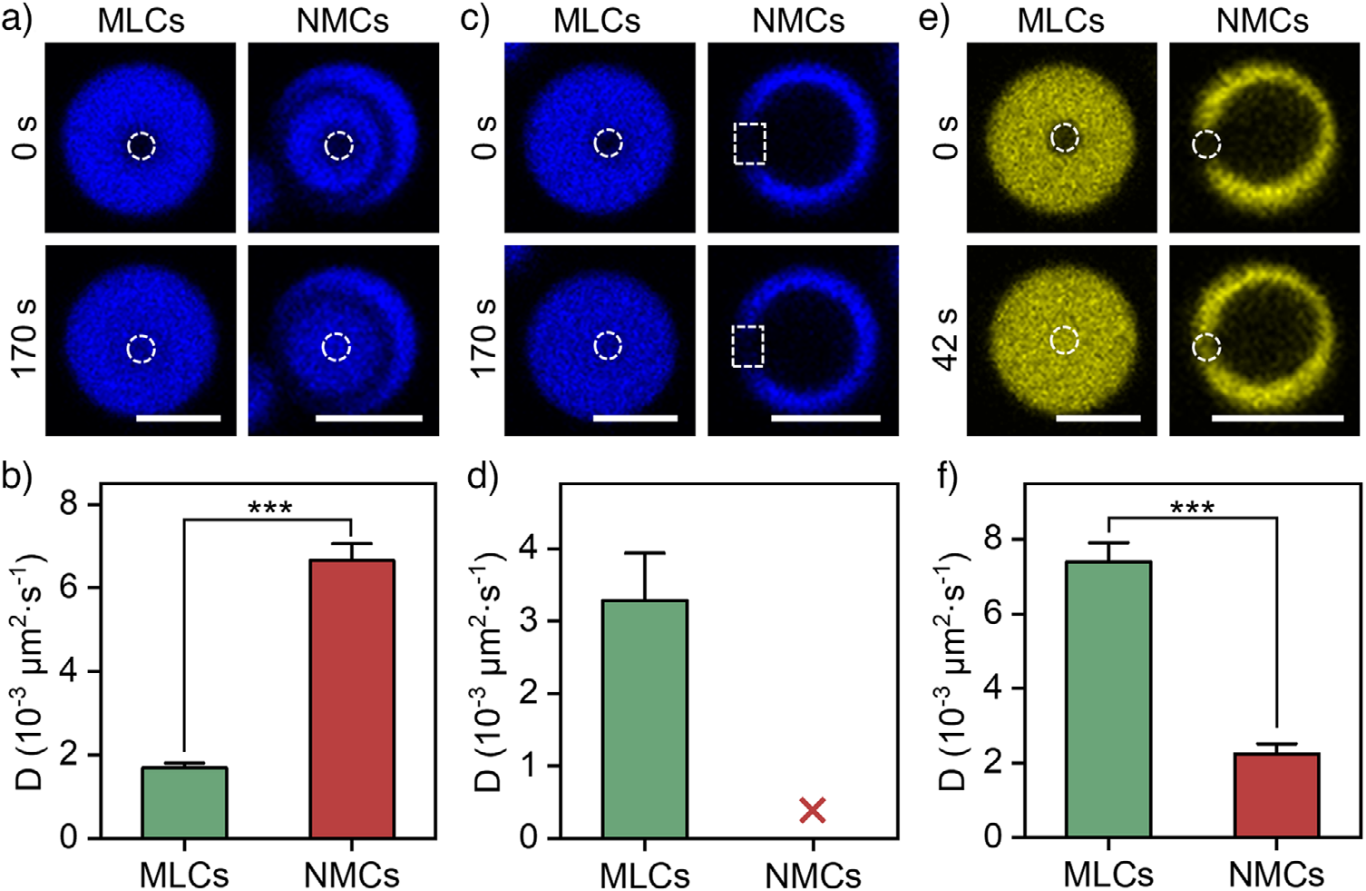
Fluidity study of coacervate components via FRAP. a)–e) Time series of CLSM images after photobleaching in MLCs and NMCs using different dye‐labeled components and corresponding calculated diffusion coefficients (*D*) for each component a) and b): Cy5‐PDDA; c) and d): Cy5‐PAH; e) and f): TNP‐ATP) (data are mean ± SD (*n* = 3, ***p < 0.001)). Scale bar: 5 µm.

As demonstrated in the preceding section, a charge reversal of the coacervate component induces a morphology transition from MLCs to NMCs within an acid environment. To further understand the impact of this transition, herein, we investigate the fluidity changes of the three coacervate components. Fluorescence recovery after photobleaching (FRAP) is performed on both MLCs and NMCs to assess component mobility. As shown in Figures 3a,d and S12, following photobleaching of a small circular area, both systems exhibit fluorescence recovery within 170 s. However, in NMCs, the inner Cy5‐PDDA part displays significantly enhanced fluidity, with an increase in the diffusion coefficient from ∼0.0017 to ∼0.0067 µm^2^·s^−1^. In contrast, Cy5‐PAH exhibits no fluorescence recovery after bleaching (Figures 3c,d and S12), indicating that it acquires a gel‐like property upon transition. Similarly, the diffusion coefficient of TNP‐ATP (2′,3′‐O‐(2,4,6‐trinitrophenyl) adenosine 5′‐triphosphate, a fluorescent analogue of ATP) decreases from ∼0.0074 µm^2^·s^−1^ in MLCs to ∼0.0022 µm^2^·s^−1^ in NMCs (Figures 3e,f and S12). Notably, this elimination or reduction in mobility for both PAH and ATP is consistently observed in NMCs prepared with weight ratios of 6/(1:1) and 6/(2:1), suggesting that the phenomenon is robust across PAH‐rich/ATP‐rich phases with different compositions (Figures S13–S16). These variations in fluidity can be attributed to the heterogeneous redistribution of coacervate components. Compared to MLCs, the PDDA‐rich/ATP‐poor phases (both inside and outside of NMCs) contain fewer polyanions, leading to a more “dilute” coacervate state with weaker electrostatic interactions, thereby increasing PDDA mobility. On the other hand, the circular PAH‐rich/ATP‐rich phase exhibits stronger electrostatic interactions, regardless of the compositional variations (Figures S13–S16). Following this phase transition, the PAH‐rich/ATP‐rich domain in NMCs demonstrates a significantly higher packing density than in MLCs. This densification likely leads to the increased entanglement and associative interactions between PAH and ATP, forming a tightly interconnected network that resists flow and deformation. These combined effects substantially reduce or eliminate the diffusional mobility of both ATP and PAH within this phase. Figure S19 shows that this high packing density smoothly correlates with the limited diffusion potential of the circular PAH‐rich/ATP‐rich domain in NMCs.

**Figure 4:**
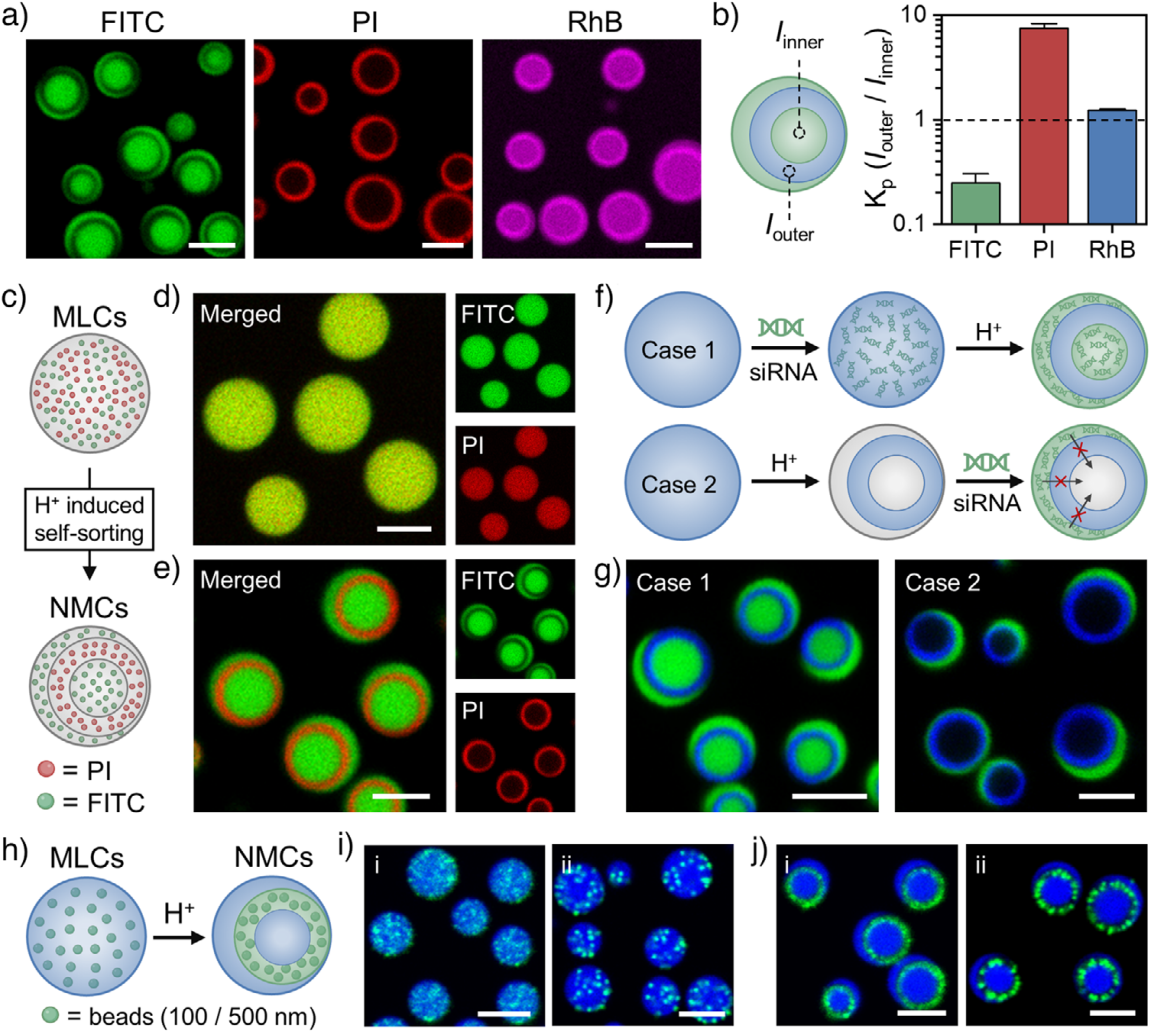
Diverse sequestration behavior of NMCs after pH‐triggered transition process at pH 6.2. a) Representative CLSM images of NMCs after incubation with FITC (anionic, green), PI (cationic, red), and RhB (zwitterionic, pink). b) Partition coefficient (*K*p) of three molecules in NMCs as determined by the ratio of fluorescent intensity in the two phases (data are mean ± SD, *n* = 5). c)–j) Schematic illustration and representative CLSM images showing the c)–e) self‐sorting behavior toward PI and FITC in this transition process (green: FITC; red: PI), f), g) siRNA sequestration behavior in the two different cases (green: siRNA; blue: Cy5‐PAH), and h)–j) spatial redistribution of polystyrene beads i): 100 nm; ii): 500 nm) before and after the transition (green: fluorescent from beads; blue: Cy5‐PDDA). Scale bar: 5 µm.

After that, to evaluate sequestration behavior, we examine the uptake of small molecules with varying charge properties, including fluorescein (FITC, anionic), propidium iodide (PI, cationic), and rhodamine B (RhB, zwitterionic). All three molecules are successfully sequestered into MLCs and display homogeneous distribution due to the inherently driven noncovalent interactions (Figure S17). However, their sequestration patterns in NMCs vary significantly. As shown in Figure 4a,b, FITC preferentially localizes in the PDDA‐rich/ATP‐poor phases due to the reduced polyanion content in these regions (partition coefficient (*K*p) ∼0.2). PI is primarily encapsulated within the PAH‐rich/ATP‐rich phase (*K*p ∼7.5), suggesting that this phase provides a more negatively charged environment. Meanwhile, RhB is sequestered into both PDDA‐rich/ATP‐poor and PAH‐rich/ATP‐rich phases, with slightly stronger affinity toward the latter (*K*p ∼1.2).

In light of the distinct encapsulation behaviors exhibited by the low‐molecular‐weight cargos in MLCs and NMCs, the influence of low‐concentration cargos (dye molecules, biomacromolecules, beads) on the pH‐dependent charge‐reversal phase transition process from MLCs into NMCs is further investigated. In other words, whether the sequence of molecular interactions affects the sequestration of formed NMCs for cargos. As shown in Figure 4c, FITC and PI are first encapsulated in MLCs before triggering the phase transition. Initially, both molecules are uniformly distributed throughout the MLCs (Figure 4d). Upon acidification (pH 6.2) and subsequent transformation into NMCs, FITC, and PI segregate into different phases: i) FITC into PDDA‐rich/ATP‐poor phases; ii) PI into PAH‐rich/ATP‐rich phase (Figure 4e). This result is analogous to the direct addition of dye molecules observed in prior‐generated NMCs. This finding indicates that small‐molecule partitioning is independent of the order of application, and a self‐sorting behavior toward different small molecules in NMCs can be achieved from this transition process. However, various biomacromolecules, such as small interfering RNA (siRNA, 13.6 kDa, negatively charged at pH 6.2), horseradish peroxidase (∼44 kDa, positively charged at pH 6.2), lysozyme (14.4 kDa, positively charged at pH 6.2), and dextran (40 kDa, neutral) exhibit same order‐dependent sequestration regardless of the surface charge of cargo (Figures 4f,g and S18). Take siRNA as an example: when it is first encapsulated into MLCs before the phase transition, it partitions into both PDDA‐rich/ATP‐poor phases in the resulting NMCs (Case 1, Figure 4f,g). In contrast, when siRNA is directly introduced into pre‐formed NMCs, it localized exclusively in the outer PDDA‐rich/ATP‐poor phase, avoiding the inner phase (Case 2, Figure 4f,g). This discrepancy may be attributed to the diffusion barrier of PAH‐rich/ATP‐rich phase due to the low porosity of this stable polyelectrolyte complex phase. This phase permits the penetration of small molecules, such as FITC, but restricts the diffusion of macromolecules (≥4 kDa) (Figure S19). Beside the various sequestration behavior toward bio(macro)molecules, nano‐scaled polymeric entities also exhibit spatial relocation in this transition process. As shown in Figure 4h–j, two polystyrene beads (amine modified, mean size: 100 nm; sulfate‐modified, mean size: 500 nm) can be effectively integrated into MLCs and show random distribution within the coacervate phase. After the pH‐triggered transition, both beads are mainly redistributed in the PAH/ATP phase and excluded by the PDDA/ATP phases. This selective localization is likely due to the PAH/ATP phase´s stronger sequestration capacity, which appears to operate independently of the surface charge of the beads. Overall, these findings demonstrate that the pH‐triggered transition introduces significant variations in both fluidity and sequestration behavior. The redistribution of coacervate components alters their mobility and intrinsic properties, ultimately leading to diverse molecular partitioning patterns.

### Secondary Reconfiguration in Coacervate Droplets Triggered by Salt Concentration

**Figure 5:**
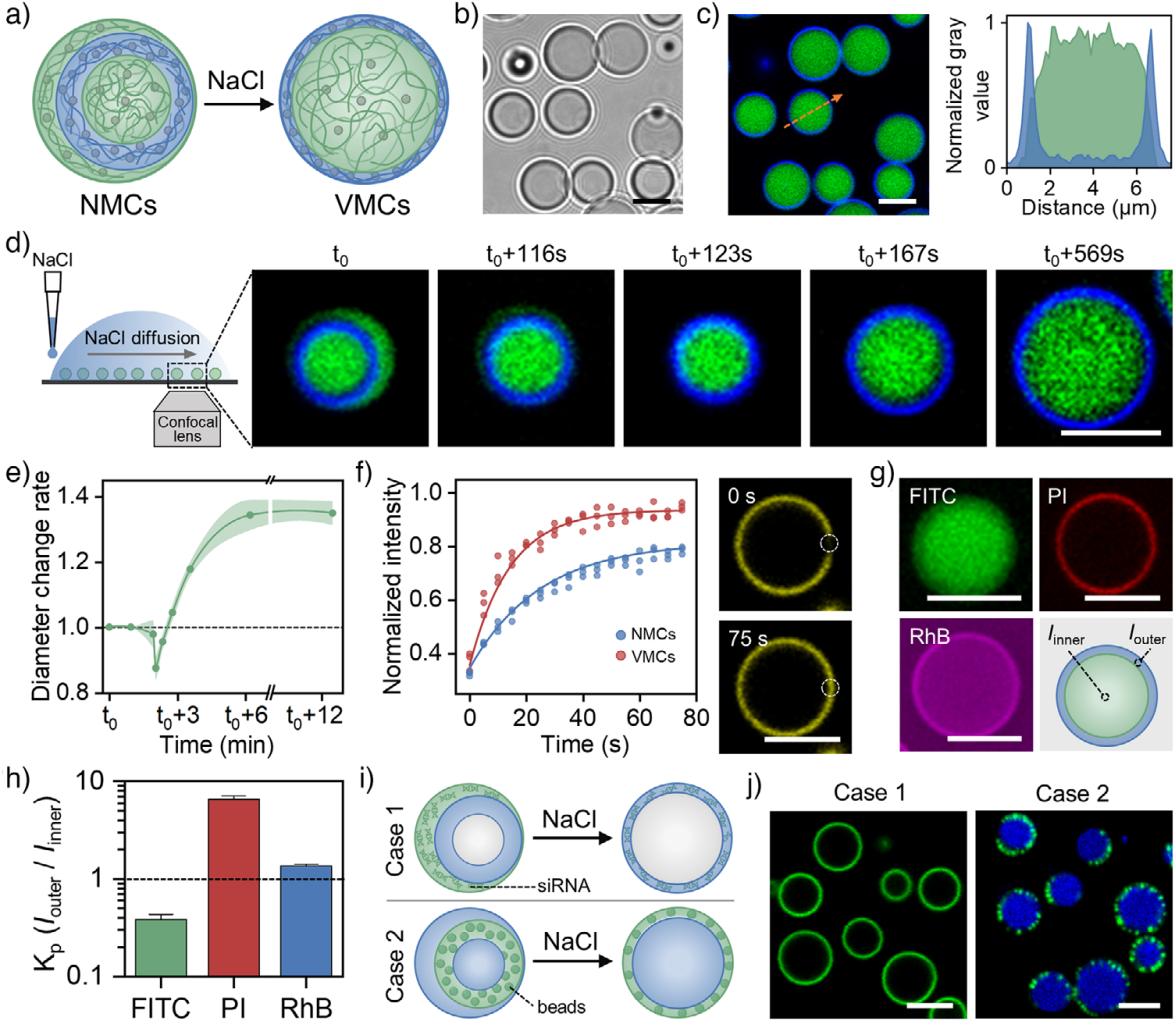
Salt concentration induced reconfiguration in multiphase coacervates from NMCs into VMCs. a)–c) Schematic illustration a) and CLSM images b): bright field; c): fluorescence field) show the formation of VMCs after addition of NaCl (50 mM), and the normalized gray value across the orange dashed line in c) (green: FITC‐PDDA; blue: Cy5‐PAH). d) Schematic illustration of the method used to observe the NaCl (50 mM) induced structural reconfiguration in‐situ and time series of CLSM images for this reconfiguration (green: FITC‐PDDA; blue: Cy5‐PAH). e) Diameter change ratio of the coacervate matrix over time in d) (data are mean ± SD, *n* = 5). f) Time series of CLSM images after photobleaching in VMCs (yellow: TNP‐ATP) and corresponding fluorescence recovery as well as fitted curves in bleached areas in NMCs and VMCs. g) Representative CLSM images of prior‐generated VMCs after incubation with FITC (green), PI (red), and RhB (pink). h) Partition coefficient (*K*p) of three molecules in VMCs as determined by the ratio of fluorescent intensity in the two phases (data are mean ± SD, *n* = 3). i) Schematic illustration and j) CLSM images show the spatial transfer of siRNA (case 1) and beads (case 2) in this NaCl (50 mM)‐induced reconfiguration (green: siRNA in case 1, fluorescent from beads in case 2; blue: Cy5‐PDDA in case 2). Scale bar: 5 µm.

The coexistence of distinct phases in multiphase complex coacervates is primarily governed by their respective critical salt concentrations.^[^ ^47^ ^]^ Following the initial transition from MLCs to NMCs under acidic condition, salt concentration as secondary stimulus is additionally used to further induce structural transformation (Figure 5a). As shown in Figures 5b,c and S20, the addition of NaCl (final concentration: 50 mM) triggers a rapid transition of NMCs into vesicle‐like coacervate structures within seconds. The coacervate structure in the second transition process is distinguished by the presence of a membrane composed of a PAH/ATP coacervate and a lumen filled with PDDA (a PDDA‐rich and ATP‐poor phase). In order to gain deeper insights into this reconfiguration process, NaCl solution is applied locally to one side of a coacervate droplet suspension. This results in the formation of a concentration gradient which effectively slows down the transformation at the opposite end (Figure 5d). This setup enables a real‐time monitoring via CLSM. As shown in Figure 5d, the diffusion of NaCl and its subsequent increase in local concentration results in the gradual dissociation of the outer PDDA/ATP coacervate phase, thereby leaving only the inner PDDA/ATP phase encapsulated by the circular PAH/ATP phase. Consequently, a decrease in the size of droplets is observed after about 2 min (Figure 5e). Instead of further dissociation, the remaining coacervate structure swells in the hypertonic environment. This results in an expansion of the circular PAH/ATP phase to approximately 1.4 times its original size, ultimately yielding vesicle‐like multiphase coacervates (VMCs). This structural reconfiguration is attributed to the differential salt tolerance of the coacervate phases in NMCs. The external PDDA‐rich/ATP‐poor phase, characterized by weaker intrinsic electrostatic interactions, dissociates in the presence of NaCl, whereas the circular PAH‐rich/ATP‐rich phase remains intact. Additionally, NaCl disrupts the electrostatic interactions within the PAH/ATP phase, leading to swelling. This transition process is also available in the presence of up to 200 mM of NaCl, indicating a high salt resistance of generated VMCs (Figure S21), and the resulting VMCs exhibit favorable structural stability over 24 h (Figure S22). Further analysis reveals that this salt concentration‐induced reconfiguration alters the fluidity of coacervate components. Again, PAH remains immobile after photobleaching (Figure S23) as demonstrated in the case of NMCs (Figure 3c,d). In opposite, ATP exhibits enhanced mobility in VMCs (Figure 5f) compared with NMCs (half‐time of recovery shortens from ∼17.7 s for NMCs to ∼8.8 s for VMCs). This enhanced mobility of ATP within the coacervate membrane can be ascribed to the weakening of ATP–PAH electrostatic interactions in the presence of NaCl (up to 200 mM).

Moreover, this NaCl‐induced transformation also influences the spatial distribution of encapsulated third‐party molecules. When three small molecules with different charge states (FITC, PI, and RhB) are introduced into VMCs, their partitioning behavior closely resembles that observed in NMCs (Figures 5g,h and S24 versus Figure 4b): FITC preferentially localizes within the PDDA‐rich lumen, exhibiting a *K*_p_ of about 0.4. PI mainly binds with the PAH/ATP coacervate membrane, with a *K*_p_ of around 6.6. RhB exhibits a slightly stronger affinity for the membrane compared to the lumen, with a *K*_p_ of approximately 1.4. However, for biomacromolecules, such as siRNA and horseradish peroxidase, which are sequestered exclusively in the outer PDDA/ATP phase in prior‐generated NMCs, the dissociation of this outer layer in the hypertonic environment (50 mM NaCl) leads to their re‐enrichment within the PAH/ATP coacervate membrane of VMCs (case 1 in Figures 5i,j and S25), indicating a spatially intra‐droplet transfer. Similarly, beads that are originally redistributed within the PAH/ATP phase of NMCs (Figure 4j) remain embedded in the PAH/ATP coacervate membrane following the NaCl‐induced transition (case 2 in Figures 5i,j and S26). These findings further corroborate the anticipated alterations in the molecular interactions of primary components in the coacervates in the presence of low‐concentration cargos during this salt concentration‐induced reconfiguration of NMCs into VMCs. This provides insights into the dynamic and versatile encapsulation behavior of multiphase coacervates.

### Tandem‐Triggered Coacervate Transition Programs the Spatial (re‐)Organization of Enzymatic Reaction

**Figure 6:**
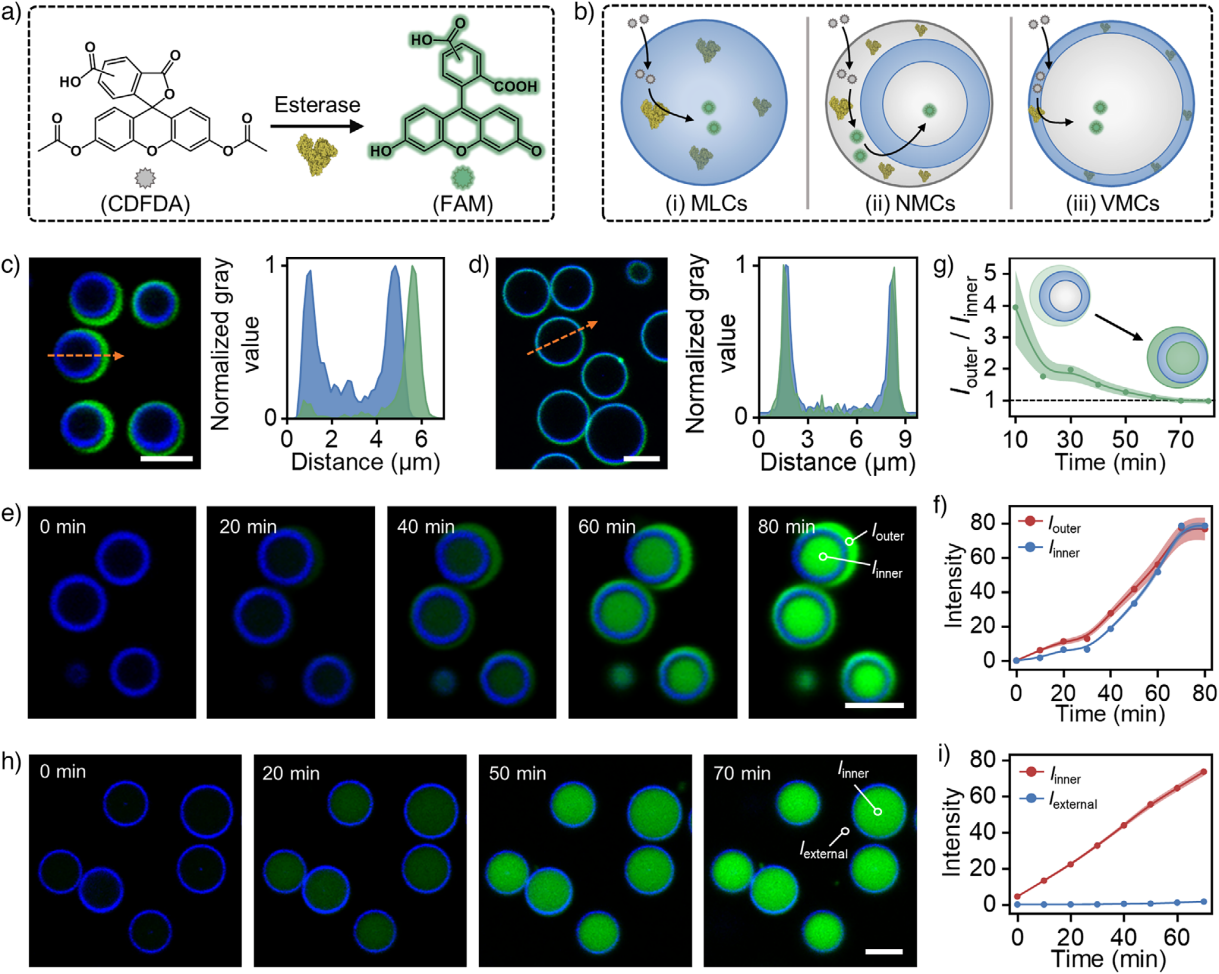
Spatial organization of enzymatic reaction in this tandem‐triggered transition. a) Schematic illustration of esterase‐mediated hydrolysis from nonfluorescent CDFDA to fluorescent FAM. b) Schematic illustration showing the different spatial organizations of enzymatic reaction in the three coacervate droplets after 1^st^ and 2^nd^ reconfiguration processes–Addition of esterase after coacervate formation processes: (i) In MLCs, both the esterase and FAM are located in the coacervate matrix; (ii) In NMCs, esterase can be sequestered exclusively in the outer PDDA/ATP phase while partially generated FAM can pass through the middle PAH/ATP phase and accumulate in the inner PDDA/ATP phase; (iii) In VMCs, esterase is combined with the PAH/ATP coacervate membrane, while FAM is excluded by the membrane itself and mainly accumulate in the lumen. c)–d) Representative CLSM images of esterase loaded NMCs c) and VMCs d) 1^st^ and 2^nd^ reconfiguration processes, as well as the corresponding normalized gray value across the dashed line in the images (green: AF 488 labeled esterase; blue: Cy5‐PAH). e) Time series of CLSM images showing the generation of green fluorescent FAM in NMCs (green: FAM; blue: Cy5‐PAH). f) Corresponding changes of fluorescence intensity and g) its ratio at two locations labeled in e) over time (data are mean ± SD, *n* = 5). h) Time series of CLSM images showing the generation of green fluorescent FAM in VMCs (green: FAM; blue: Cy5‐PAH), and (i) time‐dependent changes in fluorescence intensity (data are mean ± SD, *n* = 5). Scale bar: 5 µm.

Multiphase separation plays a crucial role in regulating the spatial distribution of enzymes, thereby influencing biological reactions.^[^ ^52^ ^]^ The tandem‐triggered transition system triggered by sequential application of acidic pH‐driven charge reversal and salt presents a versatile platform for programming enzymatic reactions spatially due to its differential sequestration properties toward bio(macro)molecules in (multi‐)phase coacervate systems. To demonstrate this capability during the reconfiguration from MLCs into NMCs and VMCs, esterase (168 kDa) is selected as a model enzyme, as it hydrolyzes the nonfluorescent 5(6)‐carboxyfluorescein diacetate (CDFDA) into a green fluorescent product, 5(6)‐carboxyfluorescein (FAM) (Figure 6a). Experimentally, esterase is encapsulated within MLCs, NMCs, and VMCs, respectively. CLSM images of this experiment series reveal that the Alexa Fluor 488 labeled esterase is homogeneously localized within MLCs, but solely present in the outer PDDA/ATP phase of NMCs after the first pH‐driven reconfiguration process, and only located within the PAH/ATP coacervate membrane of VMCs after the salt‐driven second reconfiguration process (Figures 6b–d, and S27). Upon CDFDA addition, homogenous green fluorescence is observed throughout the coacervate matrix in MLCs, indicating a time dependent in situ generation and enrichment of FAM (Figures 6b(i) and S28). In NMCs, fluorescence first occurs in the outer PDDA/ATP phase within 10 min (Figure 6e). Over time, the fluorescence signal intensifies in both the outer and inner PDDA/ATP phase (Figure 6e,f: 20 min), with the outer phase showing a higher fluorescence intensity compared to the inner phase up to 60 min (Figure 6f). Both phases reaching comparable fluorescence intensity within 80 min (Figure 6f). Quantitative analysis shows the fluorescence intensity ratio between the outer and inner PDDA/ATP phases gradually decreases from ∼4 to ∼1 over this period (Figure 6g). This redistribution of enzymatic reaction products suggests that while esterase is sequestered exclusively in the outer PDDA/ATP phase (Figure 6c), the generated FAM diffuses through the circular‐like PAH/ATP phase (Figure S29), achieving a balanced partitioning between the two PDDA/ATP compartments (Figure 6b(ii)). In VMCs, fluorescence from FAM is detected primary in the lumen (Figures 6h,i and S30), although esterase is confined to the PAH/ATP coacervate membrane (Figure 6b(iii),d). This suggests that the anionic FAM, generated within the coacervate membrane, is mainly enriched in the lumen (Figure 6biii) due to the presence of a cationic PDDA‐rich phase without major anionic (poly)electrolyte counterparts in the lumen of VMCs, which eventually leads to the formation of coacervates in the lumen of VMCs where PDDA‐rich and ATP‐poor areas exist (Figure 5c,d,f). This spatial distribution of hydrolysis products in VMCs contrasts with the patterns observed in MLCs and NMCs.

Overall, the distinct sequestration properties of the three coacervate droplets within the tandem‐triggered transition system enable diverse spatial organization of enzymatic reactions, demonstrating the potential of multiphase‐separated coacervates as tunable microenvironments for controlled reaction compartmentalization.

## Conclusion

In this study, we present a stimulus tandem‐triggered transition system that enables the transformation of membrane‐less single‐phase coacervates into structured multiphase coacervates. To achieve this, a polyelectrolyte with acid‐cleavable bonds is used as a key component in the formation of the initial single‐phase coacervates. Upon exposure to a weakly acidic environment, the charge reversal of the polyelectrolyte from negative to positive induces the redistribution of coacervate components within the initial droplets, resulting in the formation of nested multiphase coacervates with distinct structural characteristics. This transition modulates the fluidity of the coacervate components and alters the retention behavior of encapsulated bio(macro)molecules within the resulting multiphase system. In addition, the introduction of salt concentration as a second stimulus facilitates the structural reconfiguration from the nested multiphase coacervates to vesicle‐like multiphase coacervates. This reconfiguration further affects the fluidity of the coacervate components and allows for the spatial redistribution of encapsulated cargos. Additionally, the tandem‐triggered transition enables variation in the spatial programming of enzymatic reactions by exploiting the inherent differences among the three coacervate droplet states. Overall, our results highlight a strategy for precisely controlling the structural and functional transitions of coacervates through the sequential application of external stimuli: pH and salt concentration. This approach provides a versatile platform for the design of programmable and adaptive coacervate‐based protocells.

## Author Contributions

Y.Z. carried out the majority of experiments, data analysis and wrote the manuscript. B.V. performed project administration and manuscript revision. D.A. performed project administration, supervision, writing, and revision.

## Conflict of Interests

The authors declare no conflict of interest.

## Supporting information



Supporting Information

## Data Availability

The data that support the findings of this study are available in the Supporting Information of this article.

## References

[1] B. Geiger , J. P. Spatz , A. D. Bershadsky , Nat. Rev. Mol. Cell Biol. 2009, 10, 21–33. 19197329 10.1038/nrm2593

[2] J. Stelling , U. Sauer , Z. Szallasi , F. J. I. Doyle , J. Doyle , Cell 2004, 118, 675–685. 15369668 10.1016/j.cell.2004.09.008

[3] L. Lopez‐Maury , S. Marguerat , J. Bahler , Nat. Rev. Genet. 2008, 9, 583–593. 18591982 10.1038/nrg2398

[4] Z. Wang , M. Zhang , Y. Zhou , Y. Zhang , K. Wang , J. Liu , Small Methods 2023, 7, 2300042. 10.1002/smtd.202300042 36908048

[5] X. Yu , V. Mukwaya , S. Mann , H. Dou , Small Methods 2023, 7, 2300231. 10.1002/smtd.202300231 37116092

[6] I. Gözen , E. S. Köksal , I. Põldsalu , L. Xue , K. Spustova , E. Pedrueza‐Villalmanzo , R. Ryskulov , F. Meng , A. Jesorka , Small 2022, 18, 2106624. 10.1002/smll.202106624 35322554

[7] Z. Liu , W. Zhou , C. Qi , T. Kong , Adv. Mater. 2020, 32, e2002932. 32954548 10.1002/adma.202002932

[8] Z. Lin , T. Beneyton , J. C. Baret , N. Martin , Small Methods 2023, 7, 2300496. 10.1002/smtd.202300496 37462244

[9] A. B. Cook , S. Novosedlik , J. C. M. Hest , Acc. Mater. Res. 2023, 4, 287–298. 37009061 10.1021/accountsmr.2c00239 PMC10043873

[10] N. Gao , S. Mann , Acc. Chem. Res. 2023, 56, 297–307. 36625520 10.1021/acs.accounts.2c00696 PMC9910039

[11] R. Harris , N. Berman , A. Lampel , Chem. Soc. Rev. 2025, 54, 4183–4199. 40084439 10.1039/d4cs01203h PMC11907334

[12] I. B. A. Smokers , B. S. Visser , A. D. Slootbeek , W. T. S. Huck , E. Spruijt , Acc. Chem. Res. 2024, 57, 1885–1895. 38968602 10.1021/acs.accounts.4c00114 PMC11256357

[13] M. Abbas , J. O. Law , S. N. Grellscheid , W. T. S. Huck , E. Spruijt , Adv. Mater. 2022, 34, e2202913. 35796384 10.1002/adma.202202913

[14] H. Chen , Y. Bao , X. Li , F. Chen , R. Sugimura , X. Zeng , J. Xia , Angew. Chem. Int. Ed. 2024, 63, e202410566. 10.1002/anie.202410566 39103291

[15] Y. Qin , Y. S. Sohn , X. Li , R. Nechushtai , J. Zhang , H. Tian , I. Willner , Angew. Chem. Int. Ed. 2025, 64, e202415550. 10.1002/anie.202415550 39378022

[16] P. Chowdhury , B. Saha , K. Bauri , B. S. Sumerlin , P. De , J. Am. Chem. Soc. 2024, 146, 21664–21676. 39058398 10.1021/jacs.4c05624

[17] N. Modi , S. Chen , I. N. A. Adjei , B. L. Franco , K. J. M. Bishop , A. C. Obermeyer , Chem. Sci. 2023, 14, 4735–4744. 37181760 10.1039/d2sc03838b PMC10171067

[18] Q. Wang , J. B. Schlenoff , Macromolecules 2014, 47, 3108–3116.

[19] S.‐H. Oh , J. Lee , M. Lee , S. Kim , W. B. Lee , D. W. Lee , S.‐H. Choi , Macromolecules 2023, 56, 3989–3999.

[20] T. P. Fraccia , N. Martin , Nat. Commun. 2023, 14, 2606. 37160869 10.1038/s41467-023-38163-8 PMC10169843

[21] Y. Huang , X. Wang , J. Li , Y. Lin , H. Chen , X. Liu , X. Huang , ChemSystemsChem 2021, 3, e2100006.

[22] W. Mu , Z. Ji , M. Zhou , J. Wu , Y. Lin , Y. Qiao , Sci. Adv. 2021, 7, eabf9000. 34049872 10.1126/sciadv.abf9000 PMC8163073

[23] N. Martin , L. Tian , D. Spencer , A. Coutable‐Pennarun , J. L. R. Anderson , S. Mann , Angew. Chem. Int. Ed. 2019, 58, 14594–14598. 10.1002/anie.201909228 31408263

[24] Y. Zhao , S. Li , Y. Liu , C. Li , J. Zhao , Y. Ren , W. Zhao , X. Zhang , X. Cui , X. Tang , P. Ren , X. Han , Adv. Mater. 37, 2500242. 10.1002/adma.202500242 40326248

[25] T. Lu , K. K. Nakashima , E. Spruijt , J. Phys. Chem. B 2021, 125, 3080–3091. 33757284 10.1021/acs.jpcb.0c10839 PMC8020381

[26] B. Peter , A. Levrier , P. Schwille , Angew. Chem. Int. Ed. 2023, 62, e202218507. 10.1002/anie.202218507 36757674

[27] R. Merindol , S. Loescher , A. Samanta , A. Walther , Nat. Nanotechnol. 2018, 13, 730–738. 29941888 10.1038/s41565-018-0168-1 PMC6082344

[28] D. L. J. Lafontaine , J. A. Riback , R. Bascetin , C. P. Brangwynne , Nat. Rev. Mol. Cell Biol. 2021, 22, 165–182. 32873929 10.1038/s41580-020-0272-6

[29] G. A. Mountain , C. D. Keating , Biomacromolecules 2020, 21, 630–640. 31743027 10.1021/acs.biomac.9b01354

[30] Y. Chen , M. Yuan , Y. Zhang , S. Liu , X. Yang , K. Wang , J. Liu , Chem. Sci. 2020, 11, 8617–8625. 34123122 10.1039/d0sc03849k PMC8163383

[31] S. Choi , M. O. Meyer , P. C. Bevilacqua , C. D. Keating , Nat. Chem. 2022, 14, 1110–1117. 35773489 10.1038/s41557-022-00980-7

[32] Y. Ji , Y. Lin , Y. Qiao , Nat. Chem. 2025, 17, 986–996. 40467892 10.1038/s41557-025-01827-7

[33] M. Wei , X. Wang , Y. Qiao , Chem. Commun. 2024, 60, 13169–13178. 10.1039/d4cc04533e 39439431

[34] J. R. Simon , N. J. Carroll , M. Rubinstein , A. Chilkoti , G. P. López , Nat. Chem. 2017, 9, 509–515. 28537592 10.1038/nchem.2715 PMC5597244

[35] H. Karoui , M. J. Seck , N. Martin , Chem. Sci. 2021, 12, 2794–2802. 34164043 10.1039/d0sc06418a PMC8179374

[36] H. Jing , Q. Bai , Y. Lin , H. Chang , D. Yin , D. Liang , Langmuir 2020, 36, 8017–8026. 32584581 10.1021/acs.langmuir.0c01864

[37] E. R. Jimenez Granda , H. Karoui , X. Brilland , J. C. Baret , N. Martin , Chem.‐Eur. J. 2025, 31, e202501109. 40244931 10.1002/chem.202501109 PMC12133639

[38] C. Donau , F. Späth , M. Stasi , A. M. Bergmann , J. Boekhoven , Angew. Chem. Int. Ed. 2022, 61, e202211905. 10.1002/anie.202211905 PMC9828839 36067054

[39] S. L. Perry , Y. Li , D. Priftis , L. Leon , M. Tirrell , Polymers 2014, 6, 1756–1772.

[40] C. Love , J. Steinkühler , D. T. Gonzales , N. Yandrapalli , T. Robinson , R. Dimova , T.‐Y. D. Tang , Angew. Chem. Int. Ed. 2020, 59, 5950–5957. 10.1002/anie.201914893 PMC7187140 31943629

[41] S. Koga , D. S. Williams , A. W. Perriman , S. Mann , Nat. Chem. 2011, 3, 720–724. 21860462 10.1038/nchem.1110

[42] M. G. F. Last , S. Deshpande , C. Dekker , ACS Nano 2020, 14, 4487–4498. 32239914 10.1021/acsnano.9b10167 PMC7199211

[43] H. Seo , H. Lee , Nat. Commun. 2022, 13, 5179. 36056018 10.1038/s41467-022-32889-7 PMC9440086

[44] Z. Zhou , Y. Shen , J. Tang , M. Fan , E. A. V. Kirk , W. J. Murdoch , M. Radosz , Adv. Funct. Mater. 2009, 19, 3580–3589.

[45] J. Z. Du , T. M. Sun , W. J. Song , J. Wu , J. Wang , Angew. Chem. Int. Ed. 2010, 49, 3621–3626. 10.1002/anie.200907210 20391548

[46] L. Yan , W. Zheng , E. Muller , P. Carl , T. M. Hermans , G. M. Santiago , Macromolecules 2025, 58, 3635–3642.

[47] T. Lu , E. Spruijt , J. Am. Chem. Soc. 2020, 142, 2905–2914. 31958956 10.1021/jacs.9b11468 PMC7020193

[48] M. Zhu , Z. Li , J. Li , Y. Lin , H. Chen , X. Qiao , X. Wang , X. Liu , X. Huang , Nat. Commun. 2025, 16, 1783. 39971992 10.1038/s41467-025-57069-1 PMC11839979

[49] W. Mu , L. Jia , M. Zhou , J. Wu , Y. Lin , S. Mann , Y. Qiao , Nat. Chem. 2024, 16, 158–167. 37932411 10.1038/s41557-023-01356-1

[50] N. A. Erkamp , M. A. M. Verwiel , D. Qian , T. Sneideris , F. A. Spaepen , D. A. Weitz , J. C. M. van Hest , T. P. J. Knowles , Nat. Chem. Eng. 2024, 1, 430–439.

[51] P. Zhang , D. Chen , L. Li , K. Sun , J. Nanobiotechnol. 2022, 20, 31. 10.1186/s12951-021-01221-8 PMC8751315 35012546

[52] M. Prouteau , R. Loewith , Biomolecules 2018, 8, 160. 30513998 10.3390/biom8040160 PMC6316564

