# Supplementary material for: Dual‐Stimulus Programmed Multiphase Separation and Organization in Coacervate Droplets
Source: Angew Chem Int Ed Engl. 2025 Sep 7;64(40):e202512266. doi: 10.1002/anie.202512266 (PMC12462745; doi:10.1002/anie.202512266)
Supplement: Supplementary file 1 — Supporting Information [file ANIE-64-e202512266-s001.docx]

**Supporting Information**

**Dual-stimulus programed multiphase separation and organization in coacervate droplets**

*Yang Zhou,^1,2,^* Brigitte Voit,^1,2^ Dietmar Appelhans**^1,^**

^1^Division Macromolecular Chemistry, Leibniz Institute of Polymer Research Dresden, Hohe Strasse 6, Dresden 01069, Germany

^2^Organic Chemistry of Polymers, TUD Dresden University of Technology, Dresden 01062, Germany

*Corresponding authors: applhans@ipfdd.de; zhou-yang@ipfdd.de

**1. Experimental Section**

**1.1 Materials**

The following chemicals were purchased from Sigma Aldrich: adenosine 5ʹ-triphosphate disodium salt (ATP), poly(diallyldimethylammonium chloride) solution (PDDA, 100-200 kDa, 20 wt. % in H_2_O), poly(allylaminhydrochlorid) (PAH, average M_w_ 50 kDa), 2,3-Dimethylmaleic anhydride (DMMA), 2-(N-morpholino)ethanesulfonic acid (MES), fluorescein (FITC), propidium iodide (PI), Rhodamine B (RhB), latex beads (amine-modified polystyrene, fluorescent orange, aqueous suspension, mean particle size: 0.1 μm; sulfate-modified polystyrene, fluorescent orange, aqueous suspension, mean particle size: 0.5 μm), esterase from pork liver (lyophilized powder, ≥15 units/mg solid), 5(6)-Carboxyfluorescein diacetate (CDFDA), horseradish peroxidase (HRP), fluorescein isothiocyanate-labeled carboxymethyldextran (FITC-CMD; 4, 40, and 150 kDa), lysozyme from chicken egg white, fluorescein isothiocyanate-labeled dextran (FITC-Dextran, 40 kDa). 2′,3′-O-(2,4,6-Trinitrophenyl) adenosine 5′-triphosphate (TNP-ATP) (10 mM aqueous solution of pH 8) was purchased from Biolog. AF 488 NHS ester and sulfo-Cyanine5 NHS ester were purchased from Lumiprobe. Pre-treated RC membrane (MWCO: 2 kDa) was purchased from Thermo Fisher Scientific. Syringe filters (0.2 μm, Nylon; 0.8 μm, CME) were purchased from Carl-Roth. Carboxyfluorescein labeled oligonucleotide (siLuc3-FAM) (siRNA) was synthesized from Eurofins Scientific (sequence (5’->3’) [FAM](CUUACGCUGAGUACUUCGA)TT, M_w_ 13836.81 g mol^-1^). Fluorescein or Cyanine5 labeled PDDA (FITC-PDDA or Cy5-PDDA) were synthesized according to the previous work.^1^

**1.2 Characterization methods**

**Nuclear Magnetic Resonance (NMR)**

^1^H-NMR (500.13 MHz) spectra were recorded on Bruker Avance III 500 spectrometer (Bruker Biospin MRI GmbH, Ettlingen, Germany) at room temperature with D_2_O (δ = 4.79 ppm) as solvent. The chemical shifts were referenced to the corresponding solvent signals and are expressed in ppm.

**Flow cytometry**

Flow cytometry was performed with an LSRFortessa flow cytometer (Becton Dickinson, Heidelberg) using Milli-Q water as sheath fluid. The coacervates were identified by their forward versus side scatter pattern, and the side-scattered light area per particle was recorded and analyzed.

**Confocal laser scanning microscopy (CLSM)**

CLSM images were obtained on a Leica SP 8 equipped with an Argon (488 nm), a DPSS (552 nm), and a HeNe (638 nm) laser, using a 40x oil immersion objective, and acquisition processing by Leica LAS AF software (Leica Microsystems, Wetzlar, Germany). The used objectives were 10x/0.4 IMM, 20x/0.7 IMM CORR, 40x/1.25-0.7 oil. The excitation was caused by Argon laser and filters (488 nm), DPSS (552 nm), HeNe laser and filters (638 nm). Prepared samples (around 10 µL solution) were dropped onto a glass slide.

**1.3 Methods**

**Synthesis of dimethylmaleic acid modified polyacrylamide hydrochloride (PAH-DMMA)**

The PAH-DMMA was synthesized according to previously report.^2^ PAH (100 mg, 1.06 mmol NH_2_ groups) was dissolved in 5 mL NaOH (0.2 M), DMMA (315 mg, 2.5 mmol) was gradually added to the solution and the pH was kept at pH > 8.5 by adding 1 M NaOH during the reaction. The reaction was allowed to continue for an additional 3 h after the DMAA was completely added. The solution was dialyzed against water (MWCO 2 kDa, 48 h) at pH > 8.5. The product PAH-DMMA was obtained after freeze drying (107 mg, yield 55 %).

**Scheme S1**. General synthetic route of PAH-DMMA.

**Synthesis of dye labeled PAH-DMMA**

PAH (100 mg) was dissolved in 5 mL NaOH (0.2 M) and the pH was adjusted to 8.5, 5 µL of sulfo-Cyanine5 NHS ester solution (2 mg mL^-1^ in DMSO) were added, and the reaction solution was stirred for 12 h. After that, DMMA (315 mg, 2.5 mmol) was gradually added to the solution and the pH was kept at pH > 8.5 by adding 1 M NaOH during the reaction. The reaction was allowed to react for additional 3 h after the DMAA was completely added. The solution was dialyzed against water (MWCO 2 kDa, 48 h) at pH > 8.5. The product Cy5-PAH-DMMA was obtained after freeze drying.

**Preparation of PDDA/PAH-DMMA membrane-less coacervates**

***Preparation of stock solutions:*** (i) PDDA solution: 10 mg mL^-1^ in water at pH 7; (ii) PAH-DMMA solution: 10 mg mL^-1^ in water.

***Preparation of coacervate droplets:*** PDDA (10 μL) and PAH-DMMA (60 μL) were mixed at weight ratio of 1:6 to trigger the coacervation and obtaining PDDA/PAH-DMMA membrane-less coacervate microdroplets (10 mg mL^-1^). 70 μL of MES buffer (20 mM, pH 6.2) was additionally added to the formed MLCs suspension to trigger the phase transition.

**Preparation of PDDA/ATP/PAH-DMMA membrane-less coacervates**

***Preparation of stock solutions:*** (i) ATP solution: 10 mg mL^-1^ in water at pH 7; (iii) PDDA solution: 10 mg mL^-1^ in water at pH 7; (iii) FITC-PDDA solution: 10 mg mL^-1^ in water; (iv) PAH-DMMA solution: 10 mg mL^-1^ in water; (v) Cy5-PAH-DMMA solution: 10 mg mL^-1^ in water. For further CLSM study, 5 vol% (volume fraction) of FITC-PDDA or Cy5-PAH-DMMA solution was added to PDDA or PAH-DMMA solution, respectively.

***Preparation of coacervate droplets:*** ATP and PAH-DMMA (5 vol% of Cy5-PAH-DMMA contained) were mixed firstly, followed by addition of PDDA (5 vol% of FITC-PDDA contained) to trigger the formation of coacervates and obtain FITC/Cy5 labeled membrane-less coacervate microdroplets (MLCs) (10 mg mL^-1^). The formed MLCs suspension was aged for 1 h at room temperature and further diluted into 1 mg mL^-1^ by additional addition of 88 μL MES buffer (10 mM, pH 6.2) to trigger the phase transition. To maximize the extent of hydrolysis of PAH-DMMA and promote the formation of multiphase coacervates, samples used for CLSM analysis were incubated at pH 6.2 for approximately 5 hours, unless stated otherwise. MLCs with different component ratios were prepared as shown in **Table S1**.

**Table S1.** The composition of PDDA/ATP/PAH-DMMA membrane-less coacervates to prepare multi-phase coacervates through the addition of acidic buffer (**Figure 1e**).

|  |  | Polyanion/PDDA weight ratio | | |
| --- | --- | --- | --- | --- |
|  |  | 2:1 | 4:1 | 6:1 |
| ATP/PAH-DMMA weight ratio (5:1) | | 6/(10+2) | 3/(10+2) | 2/(10+2) |

Using x/(y+z) indicates the components for the preparation of MLCs, where x, y, and z also indicate the volume (μL) of PDDA, ATP, and PAH-DMMA, respectively.

**Turbidity measurements**

PDDA/PAH-DMMA based MLCs (10 mg mL^-1^) were prepared as described above. 50 μL of MLCs were transferred into cuvette followed by addition of 50 μL of MES buffer (20 mM, pH 5.6, 6.2, and 6.7, respectively). The turbidity was recorded at 500 nm and measured every 10 minutes.

**In-situ study for the pH-induced transformation process**

FITC-PDDA and Cy5-PAH-DMMA labeled MLCs (ATP/PAH-DMMA weight ratio of 5:1, polyanion/PDDA weight ratio of 6) (10 mg mL^-1^) were prepared as described above and diluted into 1 mg mL^-1^ with MES buffer (10 mM, pH 6.1) to trigger the phase transition. 150 μL of MLCs solution were transferred into a chamber slide well (18 well glass-bottom μ-slide (Ibidi, 81816)). For the in-situ study, images were captured via CLSM after the selected MLCs were transformed into multiphase coacervates (**Figure S6**).

**Fluorescence recovery after photobleaching (FRAP)**

FRAP experiments were performed on CLSM. For each sample, images were acquired before, during and after photobleaching.

For the measurement of coacervate phase fluidity, a defined circular spot with a diameter of ~1 µm was selected and bleached on Cy5-PDDA, Cy5-PAH-DMMA, or TNP-ATP labeled MLCs, NMCs, and VMCs, respectively. In particular, for Cy5-PDDA and Cy5-PAH-DMMA, 2 images were acquired before photobleaching with intervals of 1.3 s. Then, selected area was bleached 1 time with 20 % laser intensity at 638 nm with 1.3 s intervals. The post bleaching phase consist of 18 images with 10 s intervals, setting the laser intensity at 0.3 %. For TNP-ATP, 2 images were acquired before photobleaching with intervals of 1.3 s. Then, selected area was bleached 1 time with 50 % laser intensity at 488 nm with 1.3 s intervals. The post bleaching phase consist of 15 images with 3 s intervals, setting the laser intensity at 0.1 %. The fluorescence intensity inside the photobleached area was quantified using ImageJ in order to generate fluorescence recovery curves. The half time of recovery (t_1/2_) and diffusion coefficient (D) were obtained by fitting the obtained curves to first order exponential fit.^3^

**Preparation of dye labeled enzymes**

The horseradish peroxidase (HRP), lysozyme and esterase were labeled with AF488. Take esterase as an example, 50 mg of the esterase were dissolved in 10 mL of carbonate buffer (100 mM, pH 8.5), and 5 µL of AF488 NHS ester solution (5 mg mL^-1^ in DMSO) were added. Then, the reaction solution was stirred for 12 h at 4 °C, followed by the dialysis (MWCO 2 kDa) against Milli-Q water for 48 h. Any precipitates were removed by a filter (0.2 µm, Nylon filter), and after the lyophilization, AF488 labeled esterase was received. AF488 labeled HRP and lysozyme were also prepared following the same method.

**Salt concentration-induced reconfiguration in nested multiphase coacervates**

1 µL of NaCl (1 M) was added into NMCs suspension (19 µL, 1 mg mL^-1^) to reach desired NaCl concentration (50 mM) to trigger the transition from NMCs to vesicle-like multiphase coacervates (VMCs).

**Sequestration experiments**

***Preparation of stock solutions:*** (i) FITC, PI, RhB solutions: 10 µM in water, respectively. (ii) siRNA solution: 100 µM in buffer (30 mM HEPES, 100 mM KCl, 1 mM MgCl_2_, pH = 7.3). (iii) (iii) latex beads: dilute to 1 mg mL^-1^ (solid content: 0.1 wt%) in water.

Permeability of different molecules within MLCs and NMCs were performed via CLSM. No dye labeled MLCs, NMCs, and VMCs suspensions (1 mg mL^-1^) were prepared. 2 µL of FITC, PI, or RhB solution was added to coacervate suspensions (18 µL) to obtain final concentrations of 1 µM (PI, FITC, RhB, respectively), or 0.6 µL of siRNA solution was added to coacervate suspensions (19.4 µL) to obtain final concentrations of 3 µM siRNA. The CLSM images were captured after incubation of dye molecules for 10 min.

**In-situ study for salt concentration-induced reconfiguration in nested multiphase coacervates**

NMCs suspension (19 µL, 1 mg mL^-1^) was dripped onto the glass slide and kept for 10 min. Then 1 µL of NaCl (1 M) was slowly added to one side of the droplet to minimize any disturbance. The reconfiguration was captured on the opposite side of the droplet.

**Esterase-mediated enzymatic reaction in coacervate droplets**

***Preparation of stock solutions:*** (i) esterase solution: 5 mg mL^-1^ in MOPS buffer (pH 7.0, 10 mM); (ii) CDFDA solution: 0.05 mg mL^-1^ in DMSO.

***Enzymatic reaction:*** 20 µL of coacervate droplets suspension (MLCs, NMCs, and VMCs, respectively) were added into a chamber slide well (18 well glass-bottom μ-slide (ibidi, 81816)), followed by addition of 0.5 µL of esterase solution and incubated for 10 min. After that, 0.5 µL of CDFDA solution was added to trigger the enzymatic reaction and images were captured via in-situ CLSM study.

**2. Supplementary Figures**


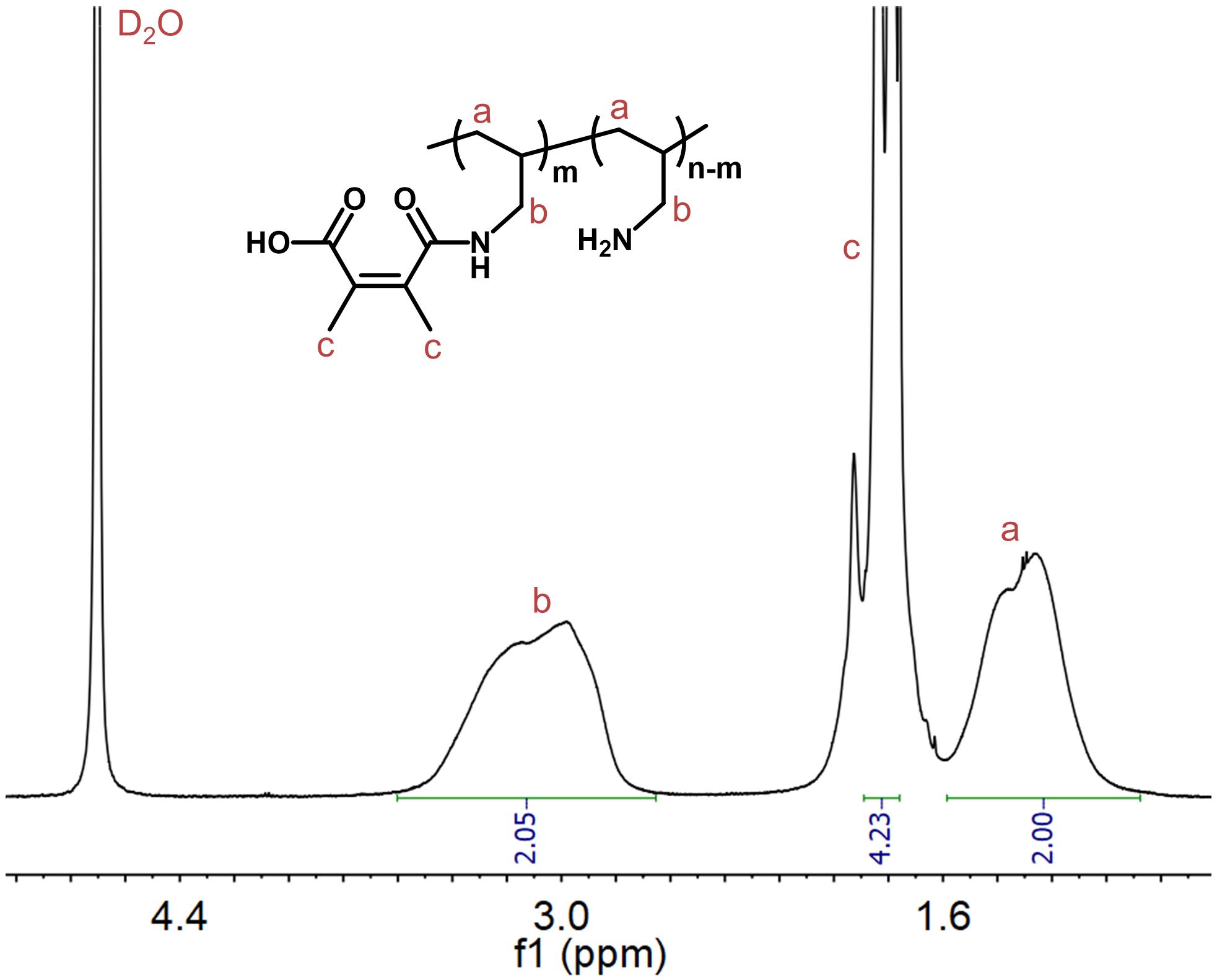


**Figure S1.** ^1^H-NMR spectrum of PAH-DMMA in D_2_O.

**Calculate the degree of substitution:**

The degree of substitution of DMAA group is determined by the presence of signals ‘a’ from 0.90 to 1.60 ppm (**-CH_2_-** of PAH backbone), the signals ‘c’ from 1.75 ppm to 1.90 ppm (**CH3-C-** of DMMA units). The integral of ‘a’ is set as 2.00 by MestReNova software (Version:6.1.0-6224), and the integral of ‘c’ is 6 when the degree of substitution of DMAA group is 100%, while the calculated integral of ‘c’ is 4.23. Therefore, the degree of substitution of DMAA group is 4.23/6 (70.5 %).


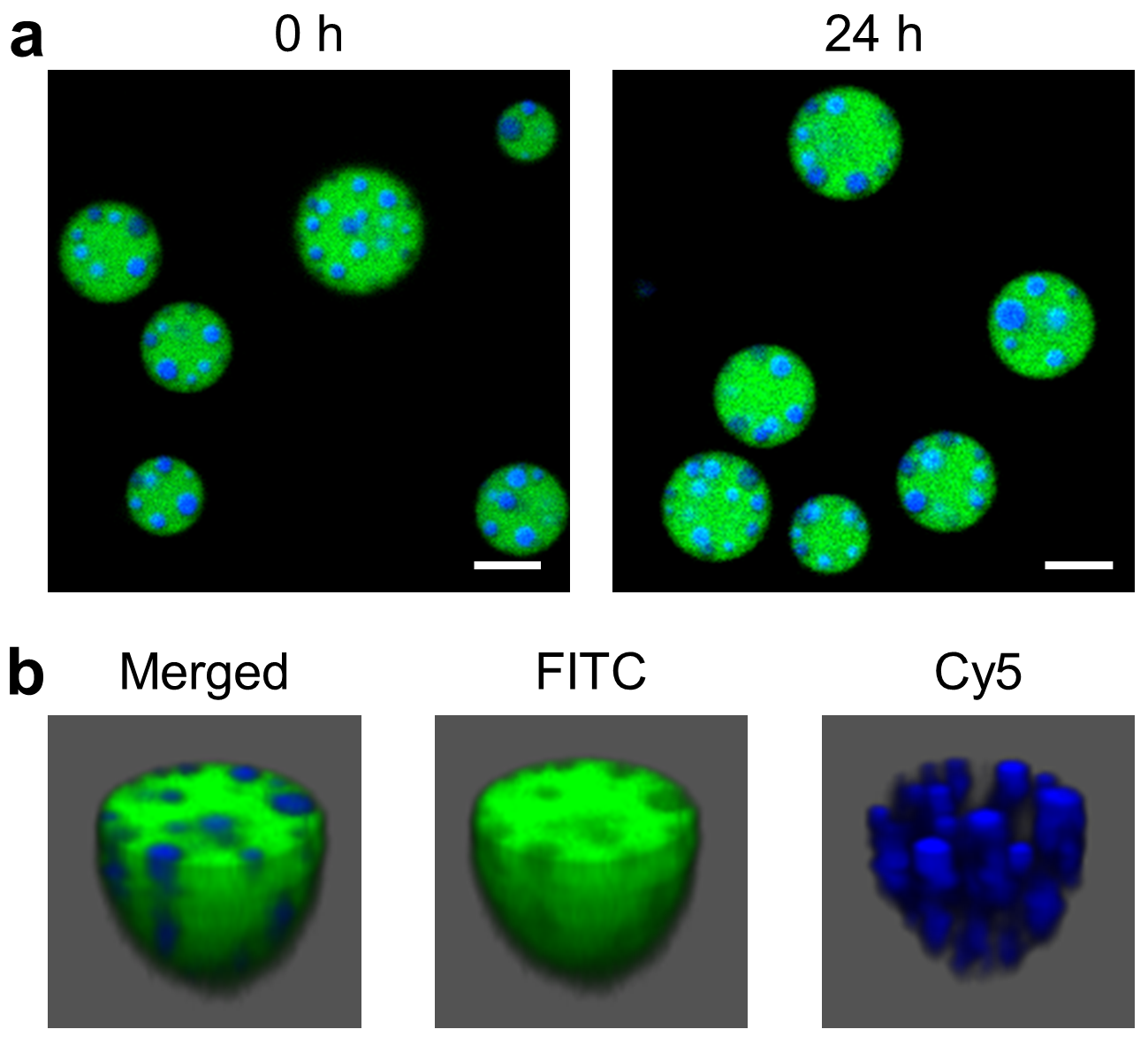


**Figure S2.** Multiphase coacervates generated from MLCs with a component ratio of 2/(5:1). (a) Stability of this multiphase coacervates and their structural integrity can be well maintained after 24 h (green: FITC-PDDA; blue: Cy5-PAH). (b) 3D image of this multiphase coacervates (green: FITC-PDDA; blue: Cy5-PAH). Scale bar: 5 μm.


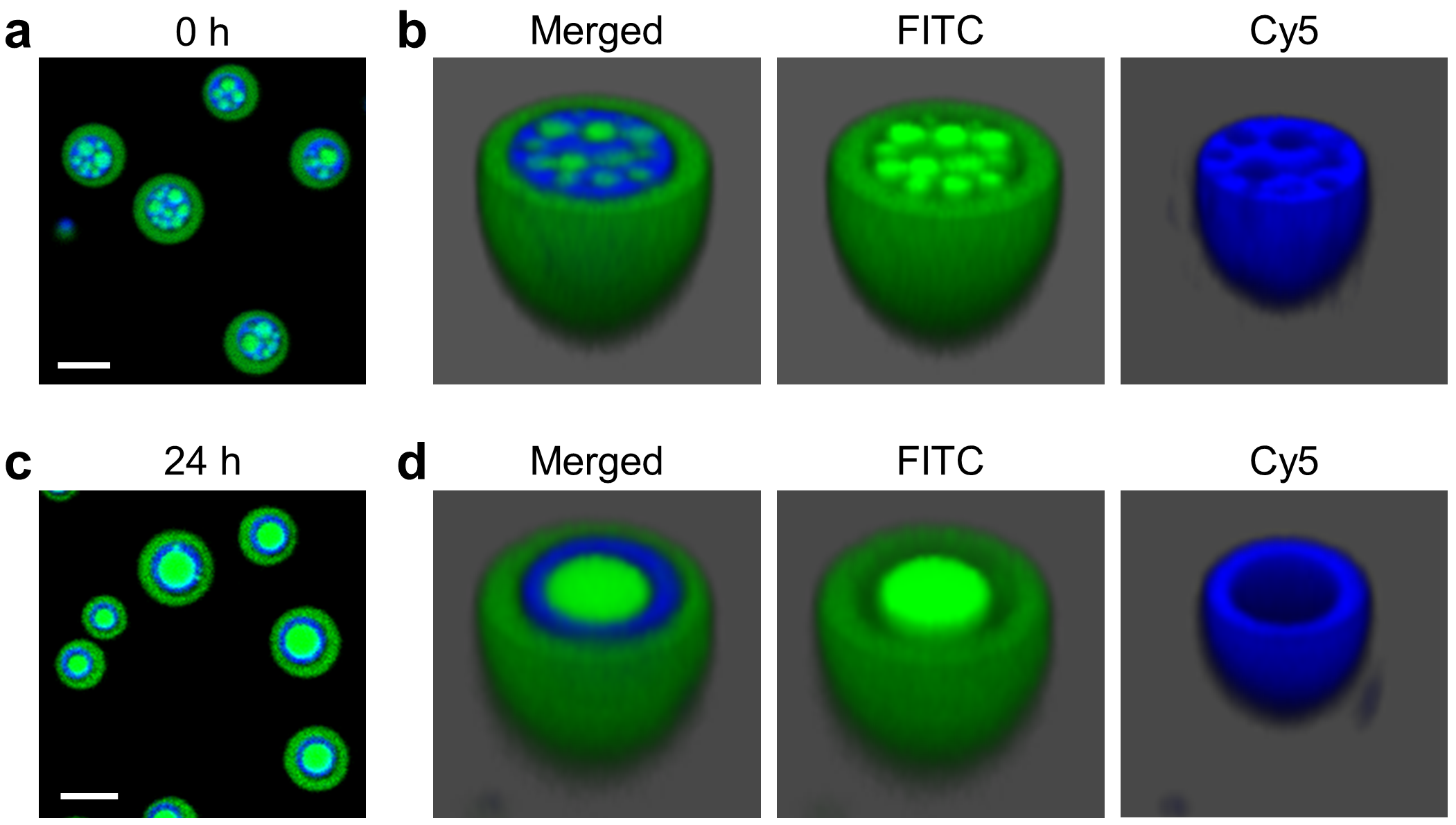


**Figure S3.** Multiphase coacervates generated from MLCs with a component ratio of 4/(5:1). (a) CLSM image and 3D reconstructed image show this multiphase coacervates at 0 h (a, b) and 24 h (c, d) (green: FITC-PDDA; blue: Cy5-PAH). As shown in this figure, these inner PDDA/ATP phases undergo fusion, ultimately forming a single, larger PDDA/ATP phase after 24 h. Scale bar: 5 μm.


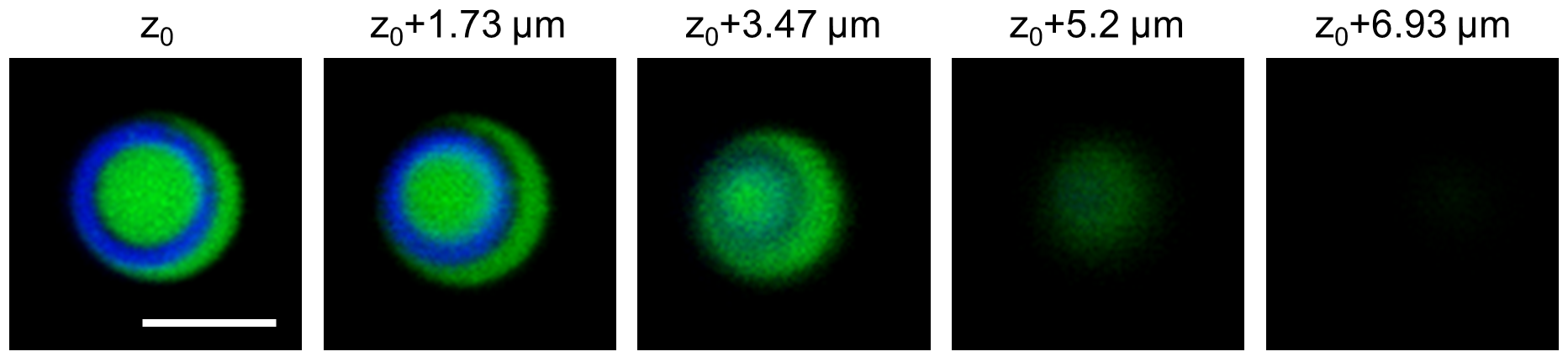


**Figure S4.** Z-axis scanning with different interval by CLSM of a single NMC as shown in **Figure 1g** (green: FITC-PDDA; blue: Cy5-PAH). Scale bar: 5 μm.


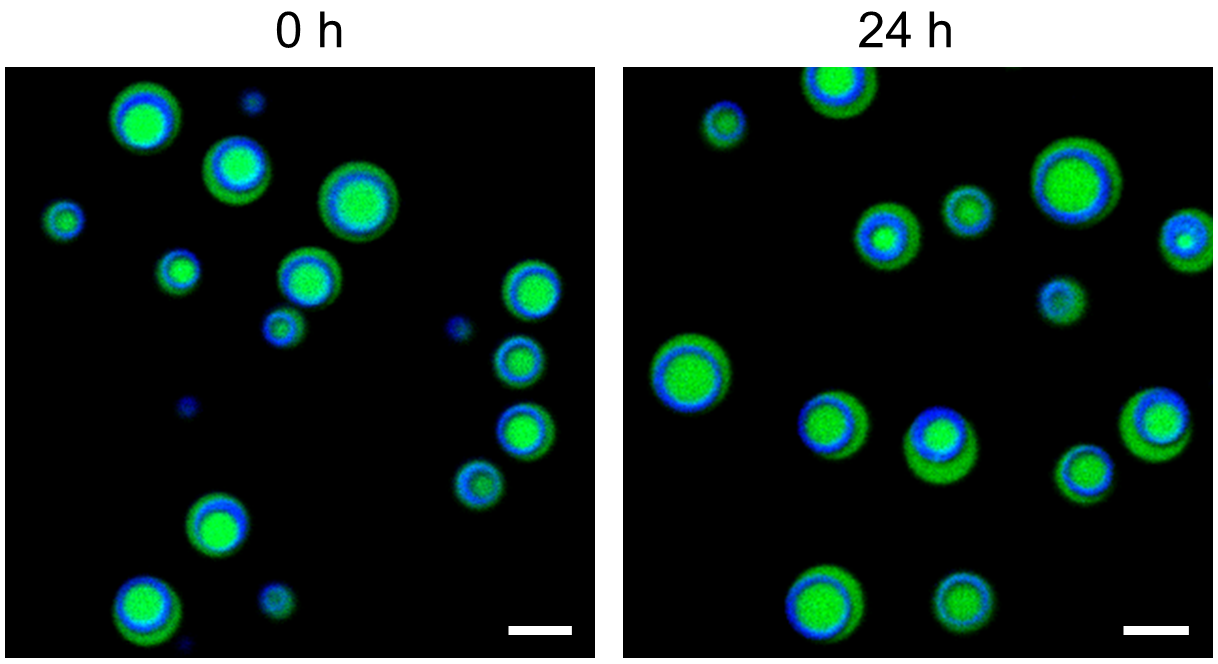


**Figure S5.** Stability of NMCs studied by CLSM: the structural integrity of VMCs can be well-kept after 24 h (green: FITC-PDDA; blue: Cy5-PAH). Scale bar: 5 µm.


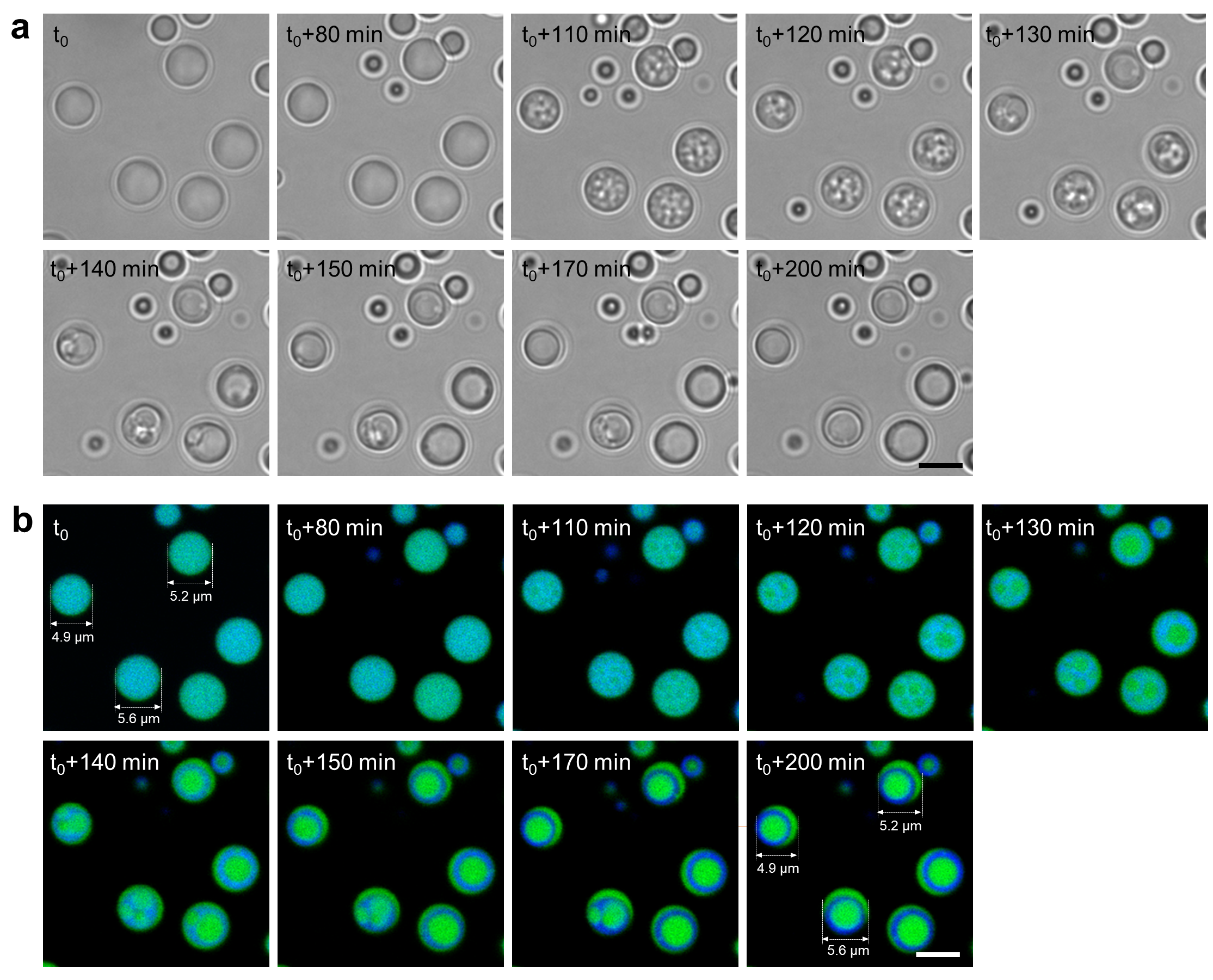


**Figure S6.** CLSM images of pH-induced transition (pH 6.2) via in-situ observation. (a) bright field and (b) fluorescence field (green: FITC-PDDA; blue: Cy5-PAH-DMMA). As shown in the figure, the coacervates gradually transform from homogeneous membrane-less coacervates into nested, structural coacervates over 200 min. During this time the coacervate size preferentially remains constant. Scale bar: 5 μm.

**
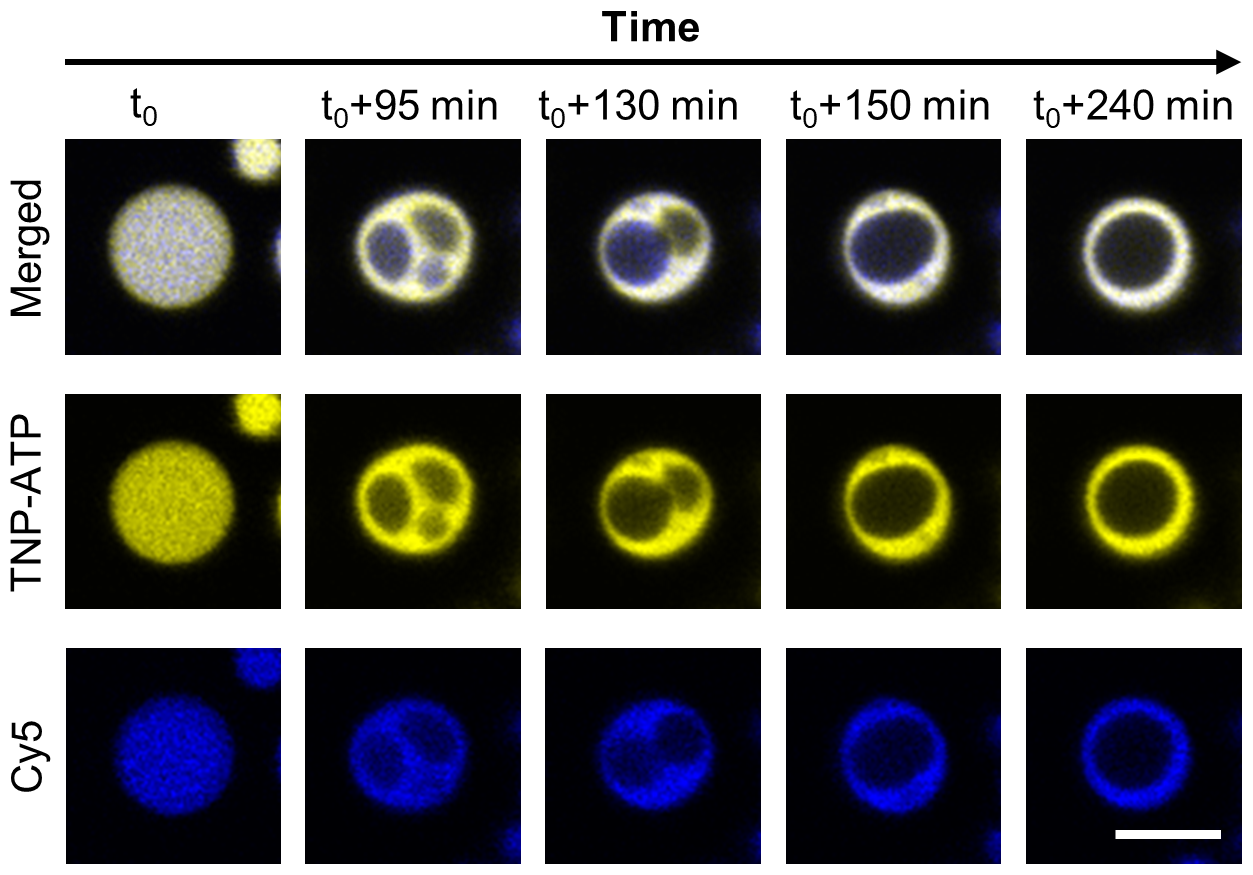
**

**Figure S7.** In-situ observation by CLSM for the location of TNP-ATP during the transition from MLCs to NMCs (yellow: TNP-ATP; blue: Cy5-PAH-DMMA). In this charge-reversal-induced transition, ATP shows the same distribution as PAH. Scale bar: 5 μm.

**
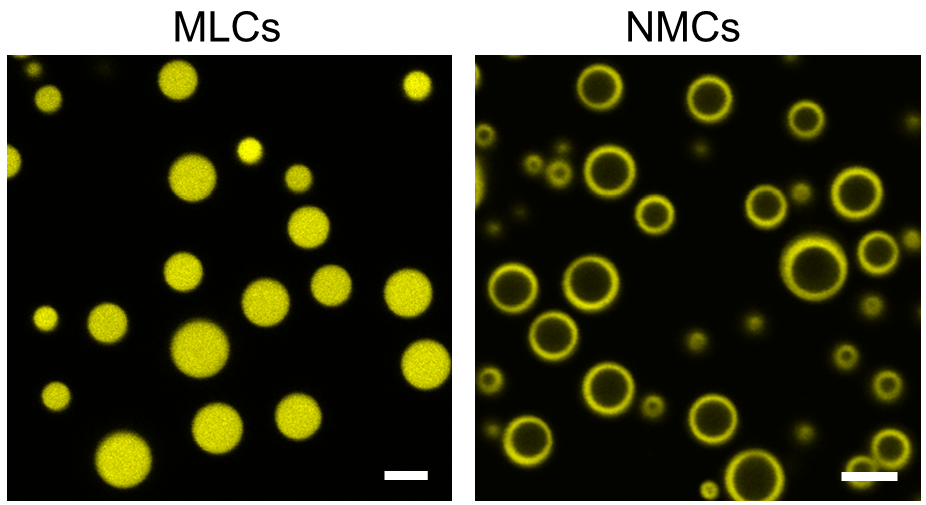
**

**Figure S8.** CLSM images showing the location of TNP-ATP (yellow) in MLCs and NMCs. TNP-ATP changes from the homogeneous distribution in MLCs to circular-like structure in NMCs. Scale bar: 5 μm.


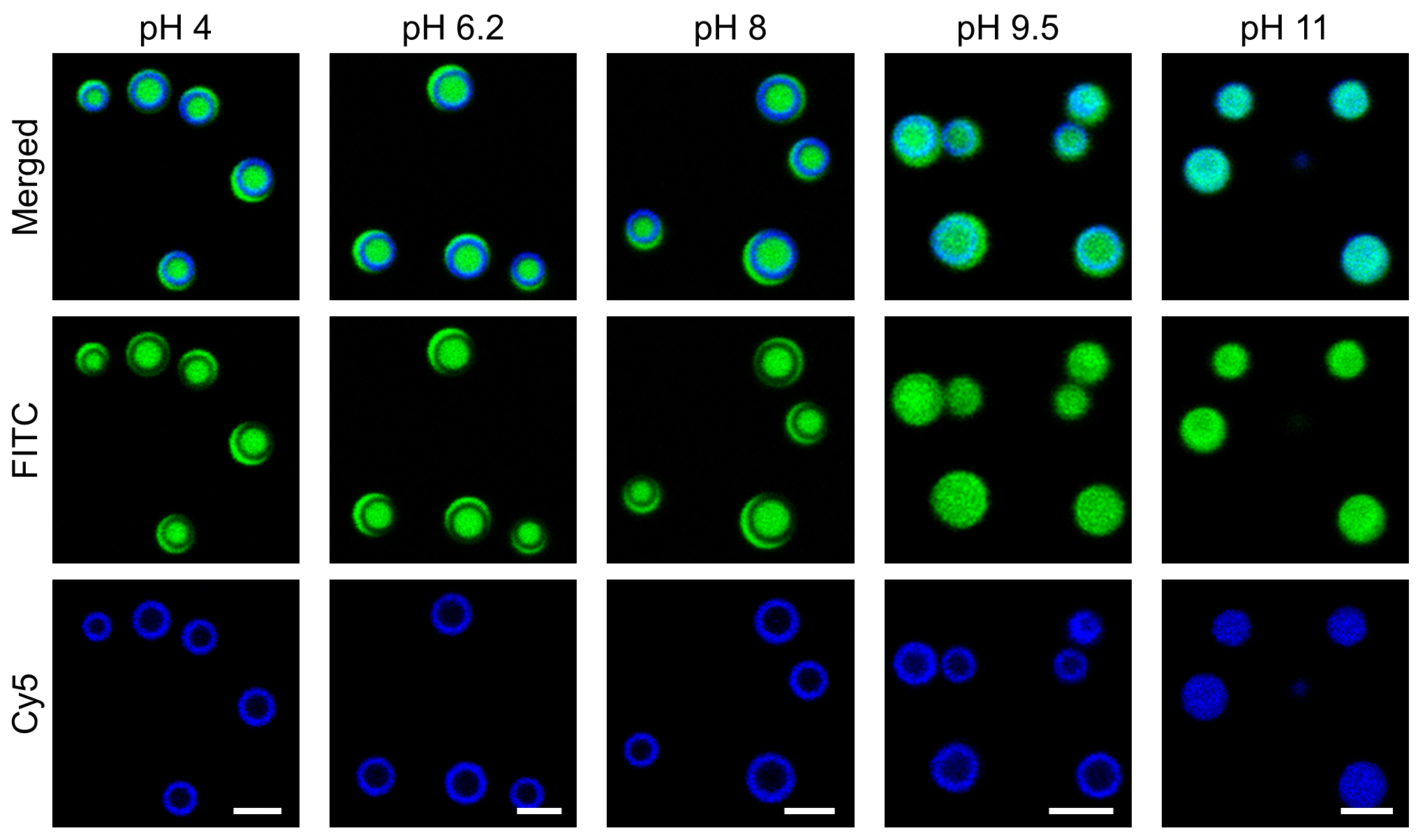


**Figure S9.** pH stability of NMCs. As shown in this figure, the nested architecture of NMCs can be maintained across a broad pH range (pH 4 to 8). However, when the pH exceeds the pK_a_ of PAH (pH 9.5 and 11), the nested structure dissipates. Briefly, at pH 9.5, the PDDA undergoes a homogeneous distribution change while PAH still retains its circular structure in the presence of ATP. At pH 11, both coacervate components are completely distributed. This can be attributed to the deprotonation of PAH, which disrupts the most electrostatic interactions between PAH and ATP. This results in the molecular reorganization between the cationic and anionic polyelectrolytes, maintaining the formation of coacervates (green: FITC-PDDA; blue: Cy5-PAH). Scale bar: 5 μm.


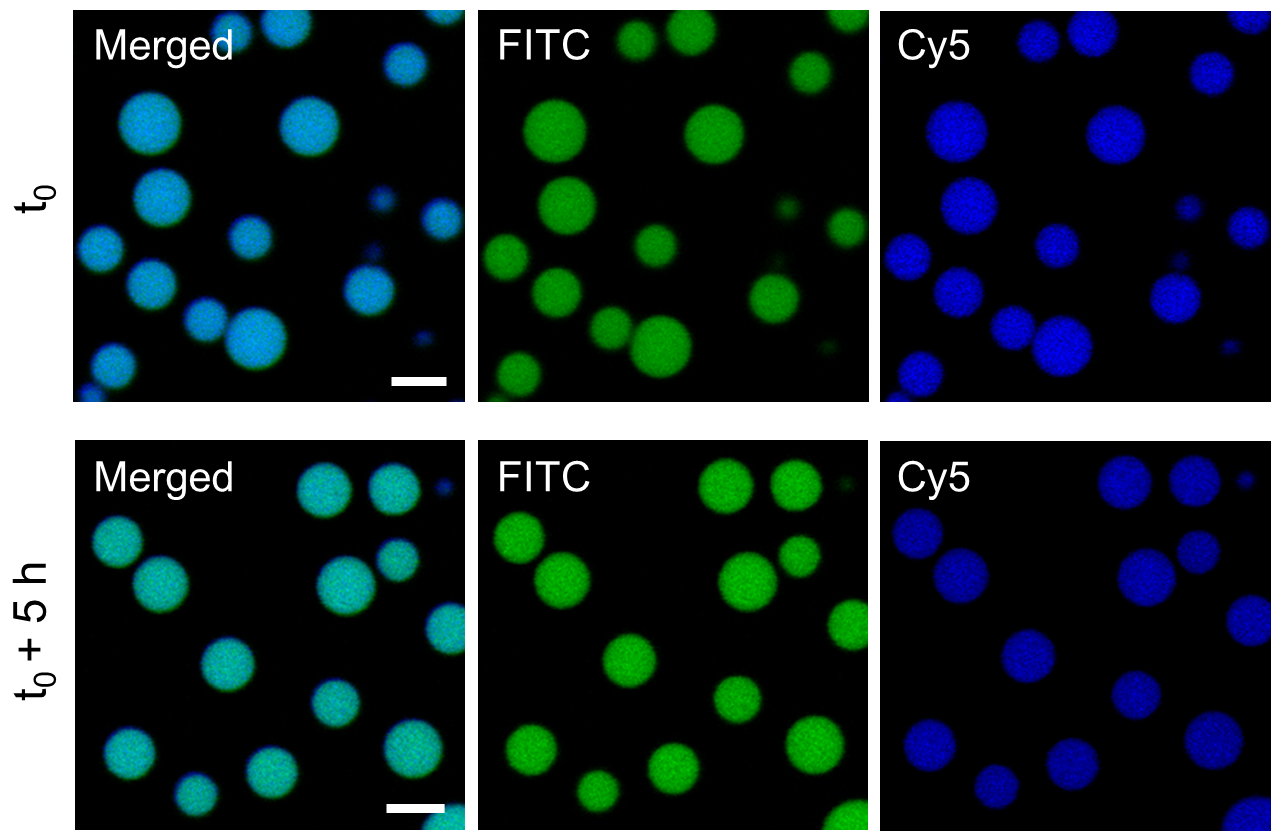


**Figure S10.** CLSM images show the MLCs after pH-induced transition process in pH 6.7 (MES, 10 mM buffer solution; green: FITC-PDDA; blue: Cy5-PAH-PMMA). After 5 h, the MLCs still keep the single-phase structure. Scale bar: 5 μm.


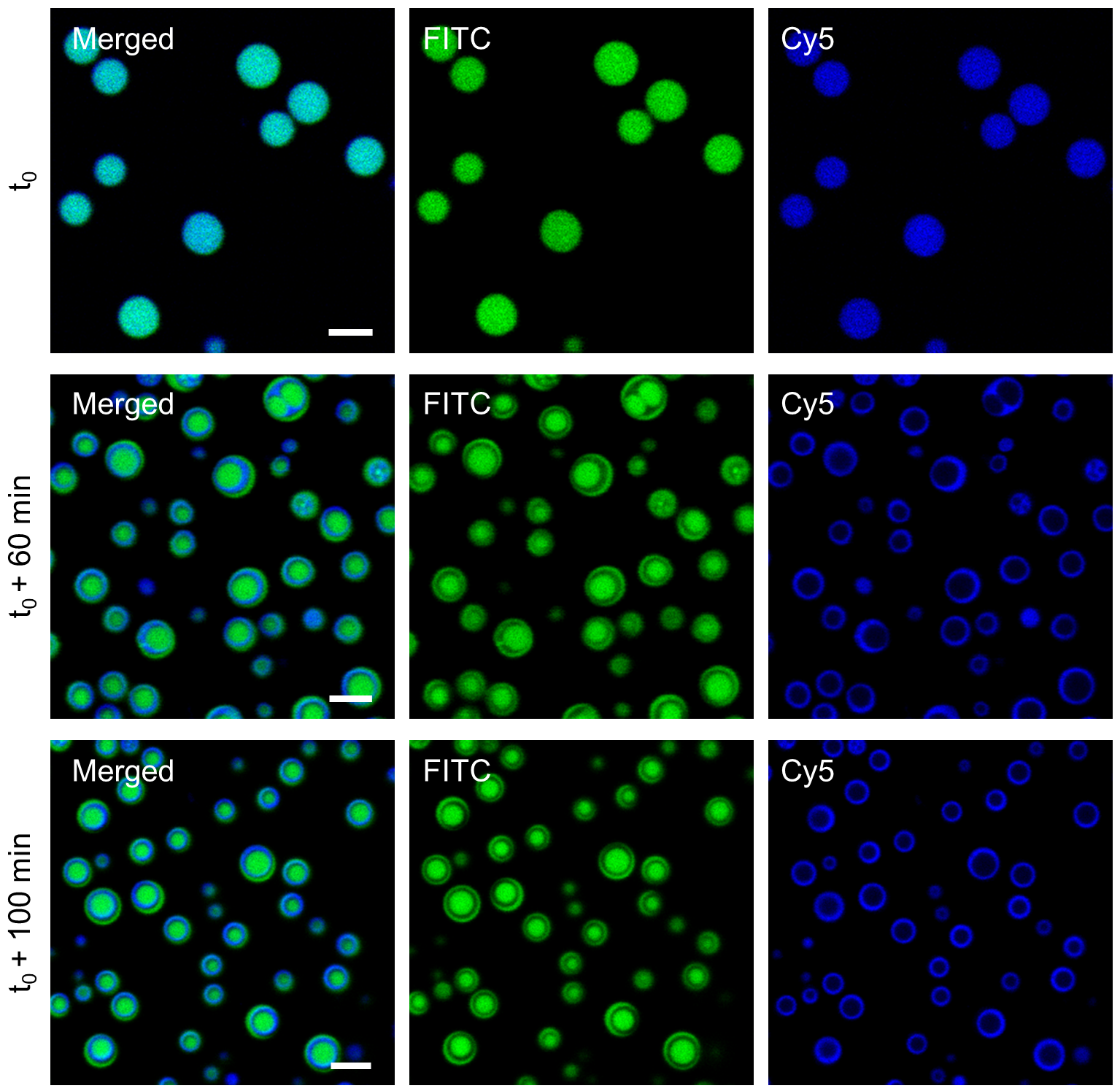


**Figure S11.** CLSM images show the MLCs after pH-induced transition process in pH 5.6 (MES, 10 mM) buffer solution (green: FITC-PDDA; blue: Cy5-PAH-PMMA). Compared to the transition triggered at pH 6.2 (**Figure 2**), a faster transition process is exhibited and the NMCs can be received within 100 min. Scale bar: 5 μm.


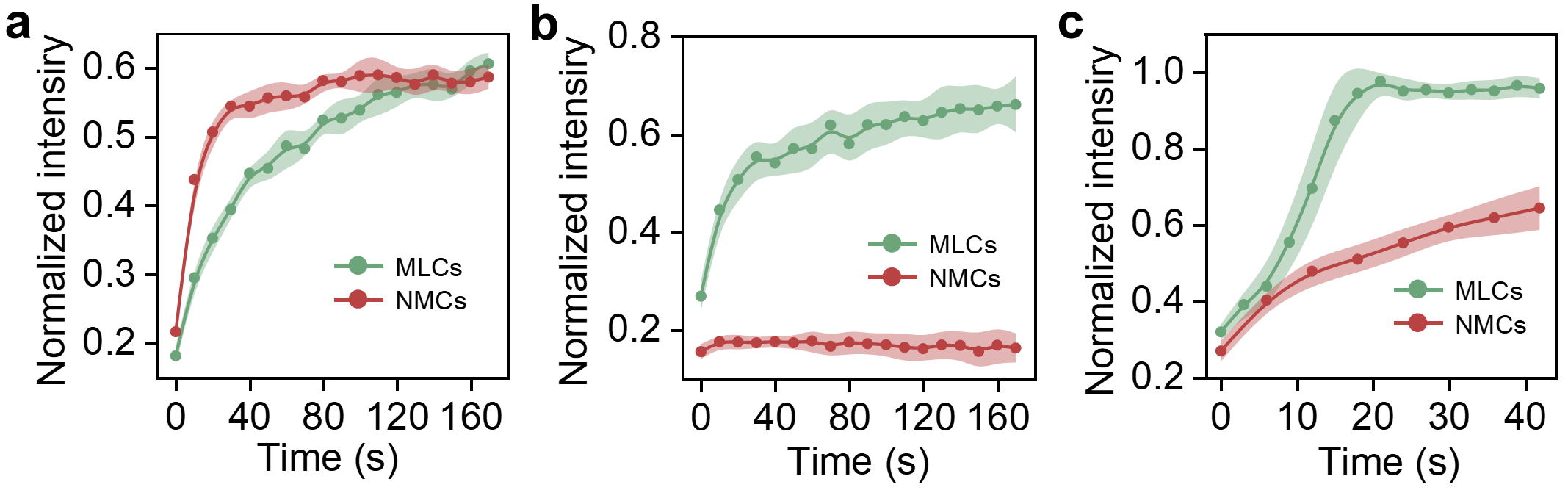


**Figure S12.** Fluidity of coacervate components in MLCs and NMCs studied by CLSM. Normalized fluorescence recovery curves for the bleached area (a: Cy5-PDDA; b: Cy5-PAH; c: TNP-ATP) shown in **Figure 3a, 3c, and 3e**. As shown in this figure, the Cy5-PDDA shows increased recovery in NMCs, Cy5-PAH exhibits no fluorescence recovery after bleaching in NMCs, and TNP-ATP shows decreased fluidity in NMCs.


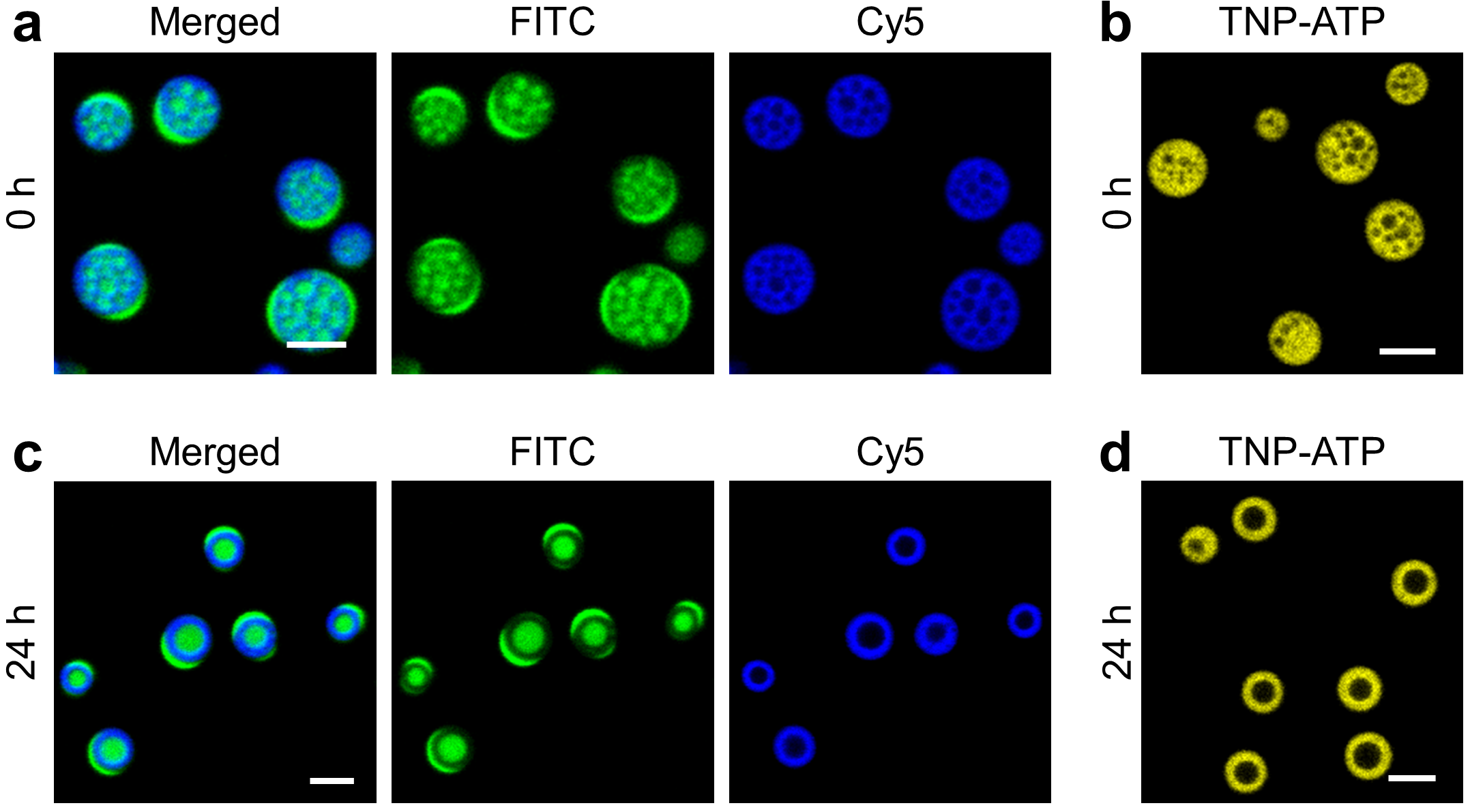


**Figure S13.** NMCs generated from MLCs with 6/(1:1) weight ratio. CLSM images show the structure of NMCs originating from MLCs with component weight ratio of 6/(1:1) (a, b) and the structure of the NMCs over the subsequent 24 h (c,d) (green: FITC-PDDA; blue: Cy5-PAH; yellow: TNP-ATP). After acidification, the generated NMCs produce a complex structure, where numerous smaller PDDA-rich/ATP-poor phases are encapsulated by the PAH-rich/ATP-rich phase, as shown in this figure. Over the subsequent 24 h, these inner PDDA/ATP domains undergo fusion, ultimately forming a single, larger PDDA-rich/ATP-poor phase. Scale bar: 5 μm.


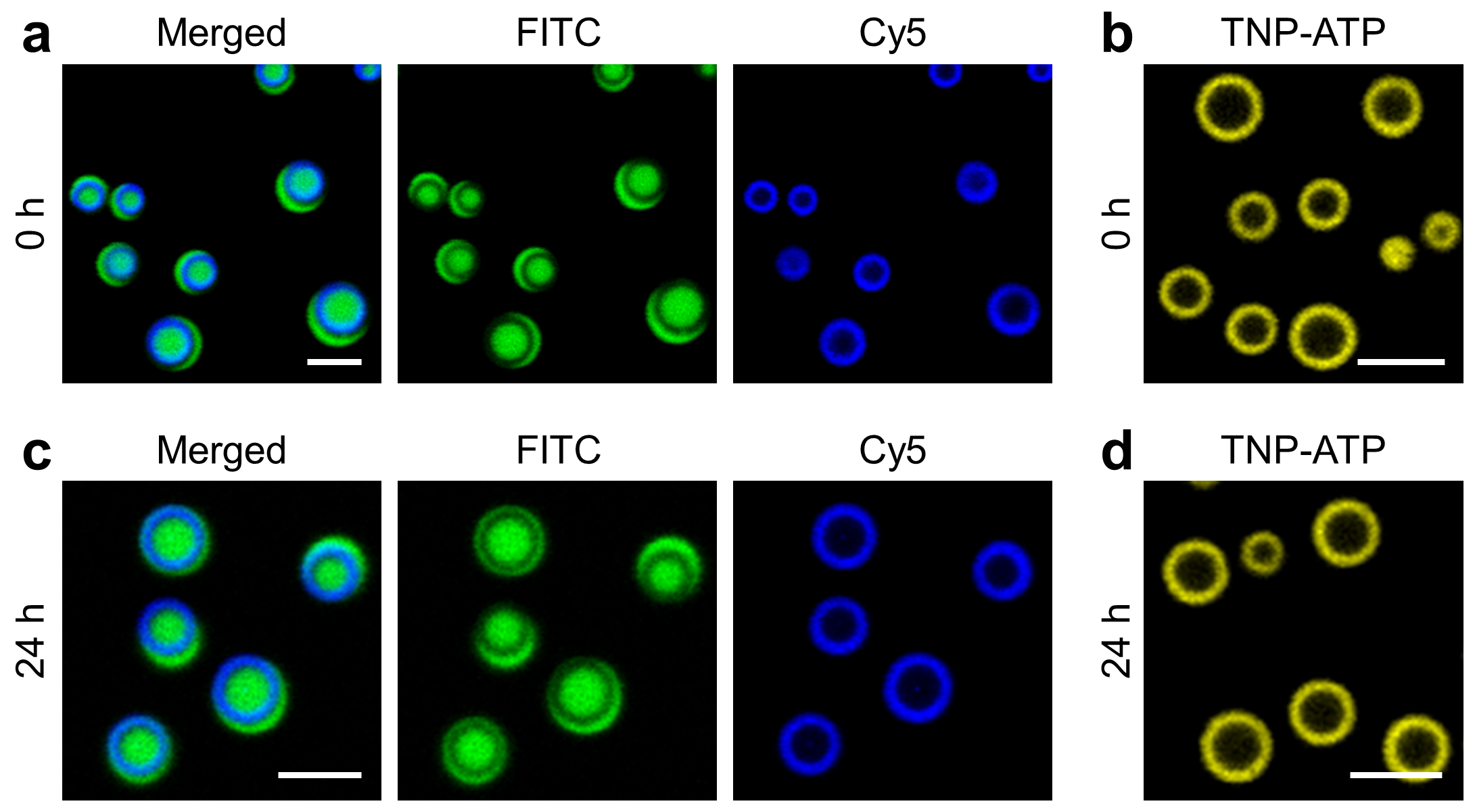


**Figure S14.** NMCs generated from MLCs with 6/(2:1) weight ratio. CLSM images show the structure of NMCs originating from MLCs with component weight ratio of 6/(2:1) (a, b) and the structure of the NMCs over the subsequent 24 h (c,d) (green: FITC-PDDA; blue: Cy5-PAH; yellow: TNP-ATP). After acidification, the generated NMCs exhibit a similar structure to NMCs generated from 6/(5:1) weight ratio (**Figure 1g**): circular-like PAH-rich/ATP-rich phase separates the PDDA-rich/ATP-poor phases into distinct internal and external regions, as shown in this figure. Moreover, the structural integrity can be well maintained after 24 h. Scale bar: 5 μm.


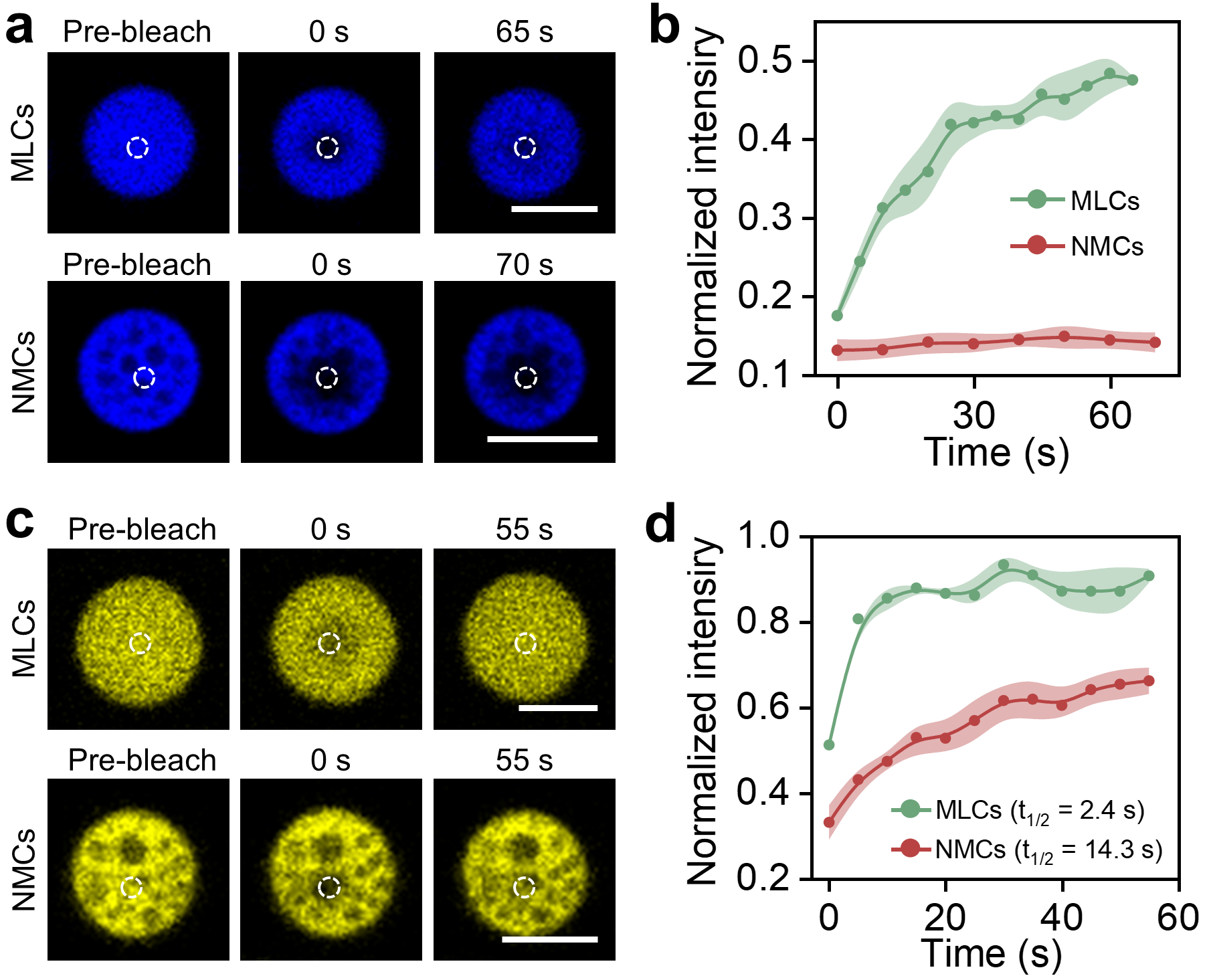


**Figure S15.** Fluidity study of components via FRAP on coacervates with weight ratio of 6/(1:1). (a-d) Time series of CLSM images after photobleaching in MLCs and NMCs using different dye-labeled components and corresponding fluorescence recovery curves in bleached areas for each component (a and b: Cy5-PAH; c and d: TNP-ATP). As shown in this figure, after the acid-triggered transition (5 h of incubation at pH 6.2), Cy5-PAH exhibits no fluorescence recovery after bleaching, and TNP-ATP shows reduced fluidity (Data are mean ± SD, n=3). Scale bar: 5 μm.


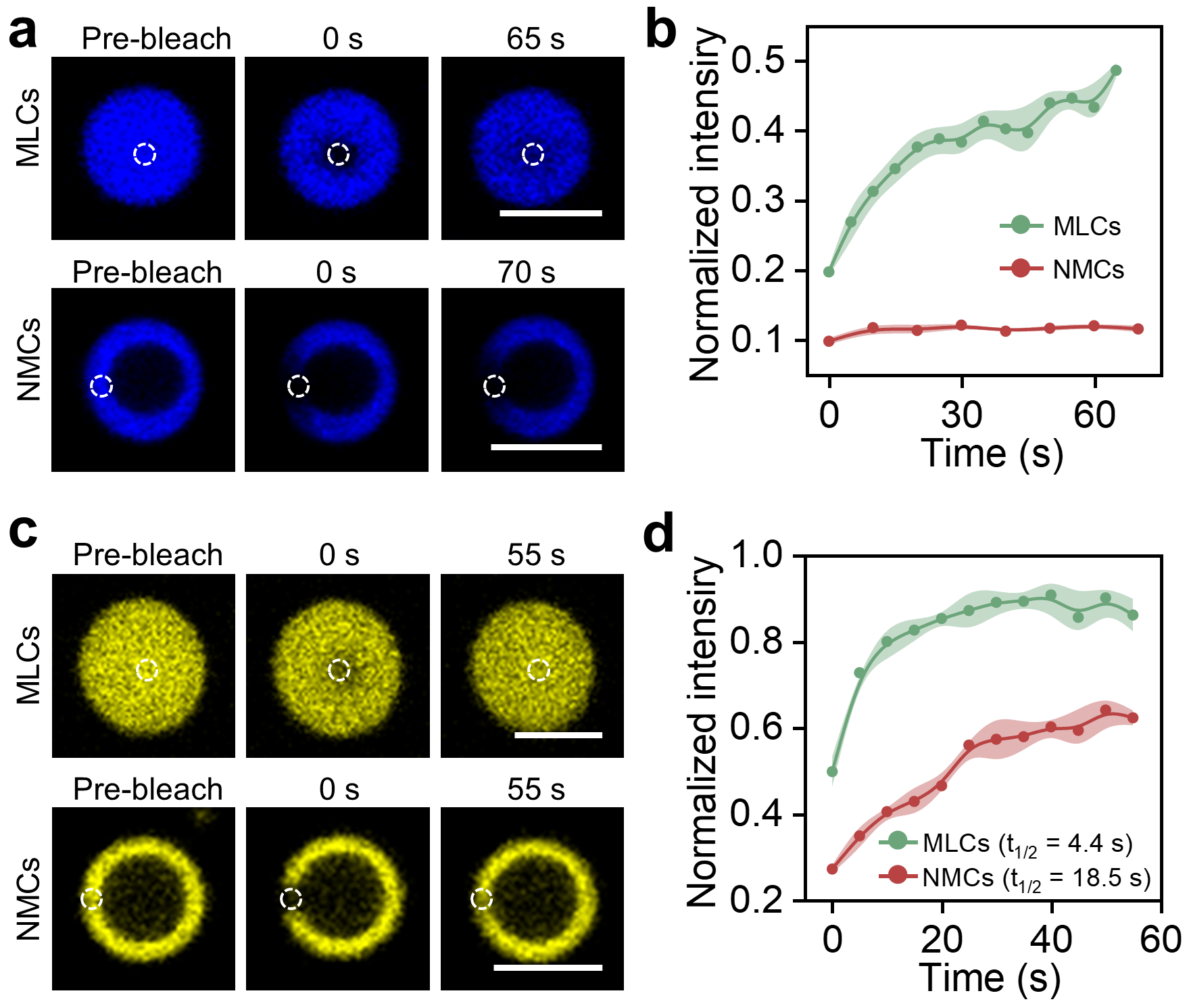


**Figure S16.** Fluidity study of components via FRAP on coacervates with weight ratio of 6/(2:1). (a-d) Time series of CLSM images after photobleaching in MLCs and NMCs using different dye-labeled components and corresponding fluorescence recovery curves in bleached areas for each component (a and b: Cy5-PAH; c and d: TNP-ATP). As shown in this figure, after the acid-triggered transition (5 h of incubation at pH 6.2), Cy5-PAH exhibits no fluorescence recovery after bleaching, and TNP-ATP shows reduced fluidity (Data are mean ± SD, n=3). Scale bar: 5 μm.


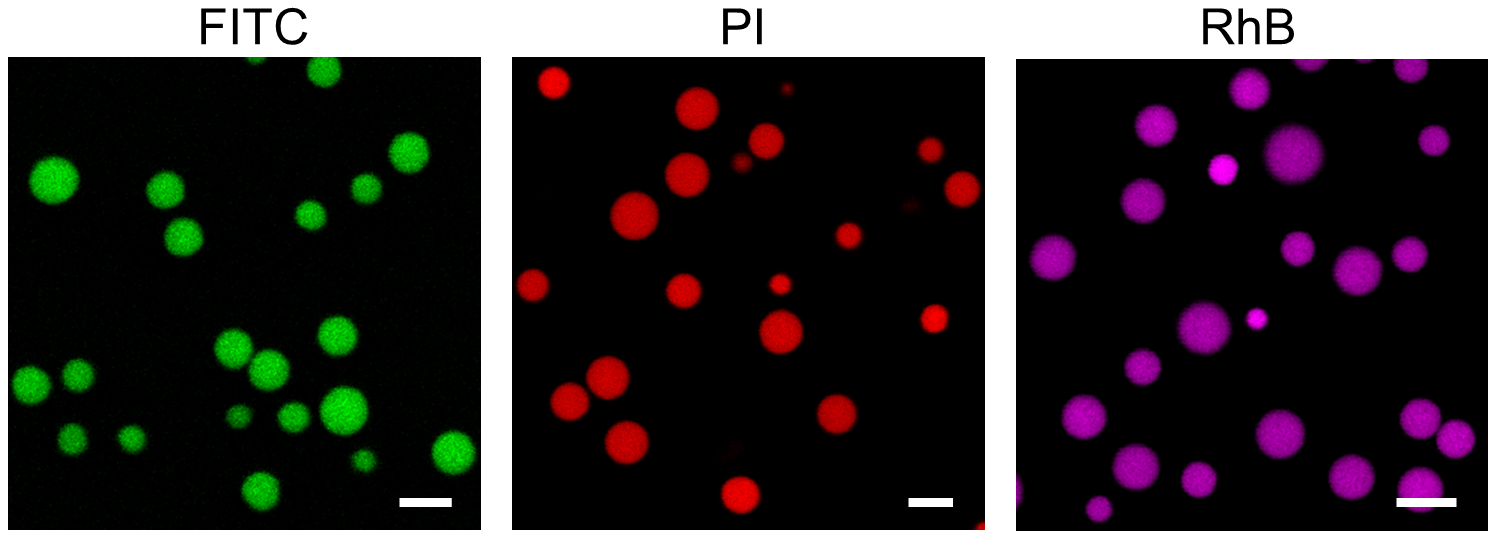


**Figure S17.** CLSM images for the sequestration behavior towards various small molecules in MLCs. All three can be sequestered into MLCs and exhibit homogeneous distribution in the coacervate phase. Scale bar: 5 μm.


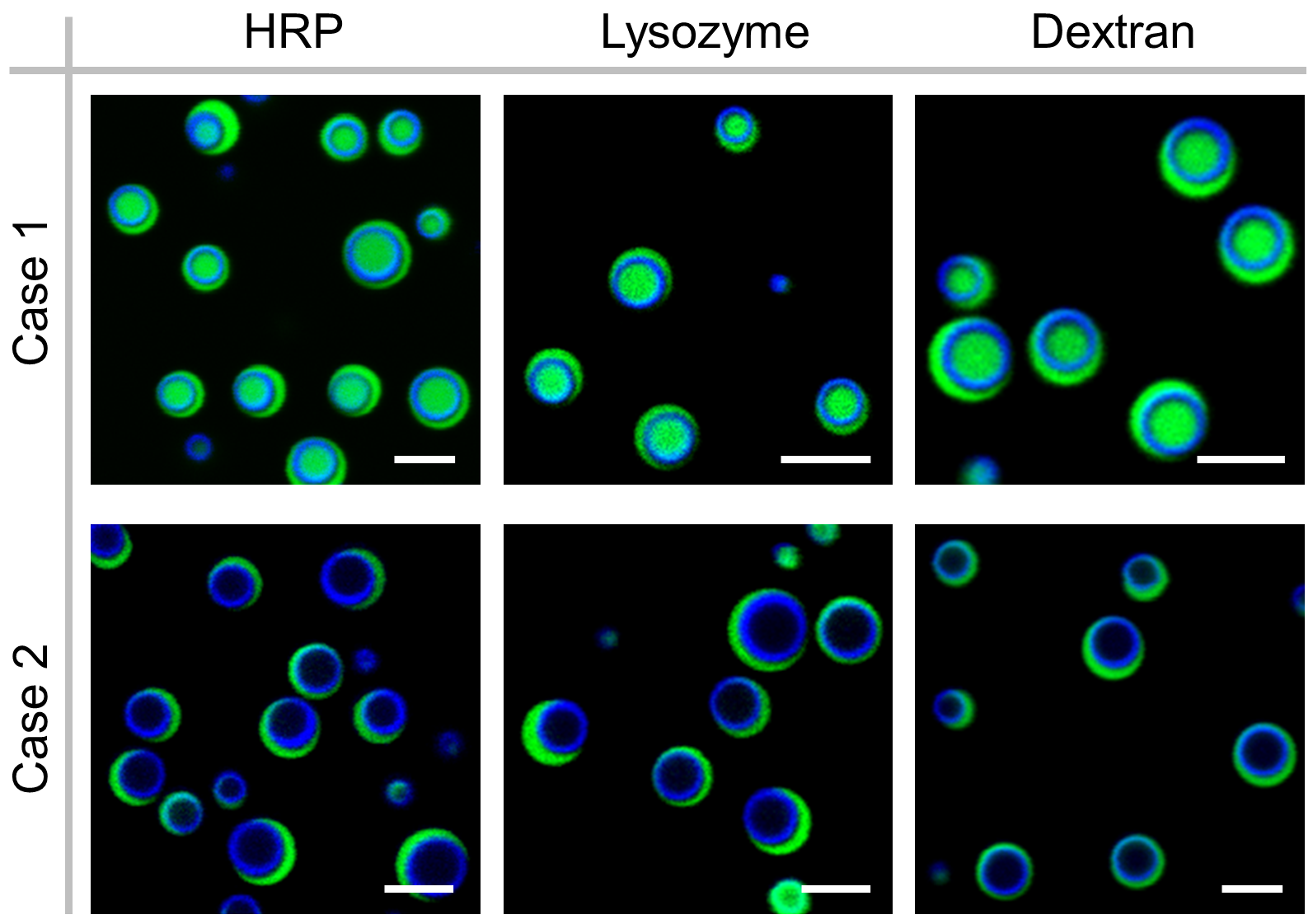


**Figure S18.** The order-dependent sequestration of various macromolecules under different approaches. If the HRP, lysozyme, or dextran (40 kDa) are encapsulated into MLCs before the pH-induced phase transition, they partition into both PDDA-rich/ATP-poor phases in the resulting NMCs (Case 1). By contrast, when these macromolecules are directly introduced into prior-generated NMCs, they localize exclusively in the outer PDDA-rich/ATP-poor phase, avoiding the inner phase (Case 2) (green: AF 488 labeled HRP, AF 488 labeled lysozyme, or FITC labeled dextran (40 kDa); blue: Cy5-PAH). Scale bar: 5 µm.


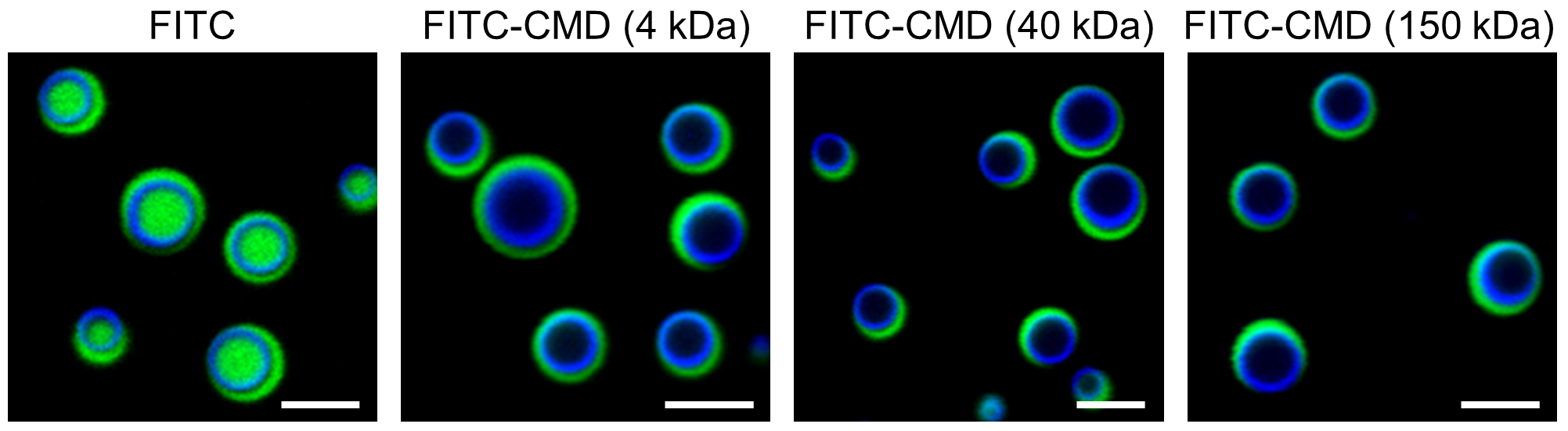


**Figure S19**. Molecular weight cutoff of the circular PAH/ATP phase. After the addition of these (macro)molecules into the established NMCs, the FITC molecule can be sequestered into both the outer and inner PDDA/ATP phases. However, FITC-CMD with different molecular weights (4 kDa, 40 kDa, and 150 kDa) can only be sequestered into the outer PDDA/ATP phase. This clearly indicates that the molecular weight cutoff of the circular PAH/ATP phase may be less than 4 kDa (green: FITC or FITC-CMD; blue: Cy5-PAH). Scale bar: 5 μm.


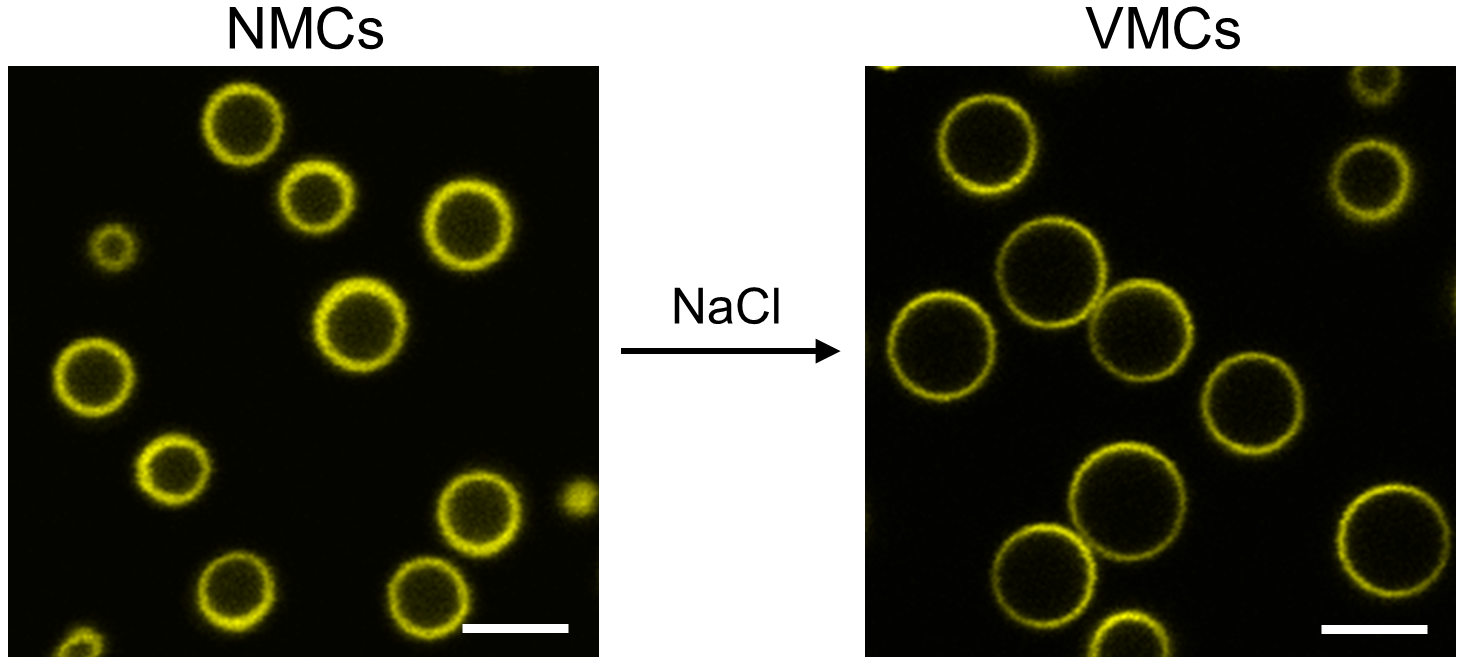


**Figure S20.** Salt-induced transition process of NMCs to VMCs. CLSM images showing the location of TNP-ATP in the coacervates before and after the NaCl (50 mM) addition. As shown in this figure, TNP-ATP (yellow) changes from the circular-like structure in NMCs to a thinner membrane in VMCs. Scale bar: 5 µm.


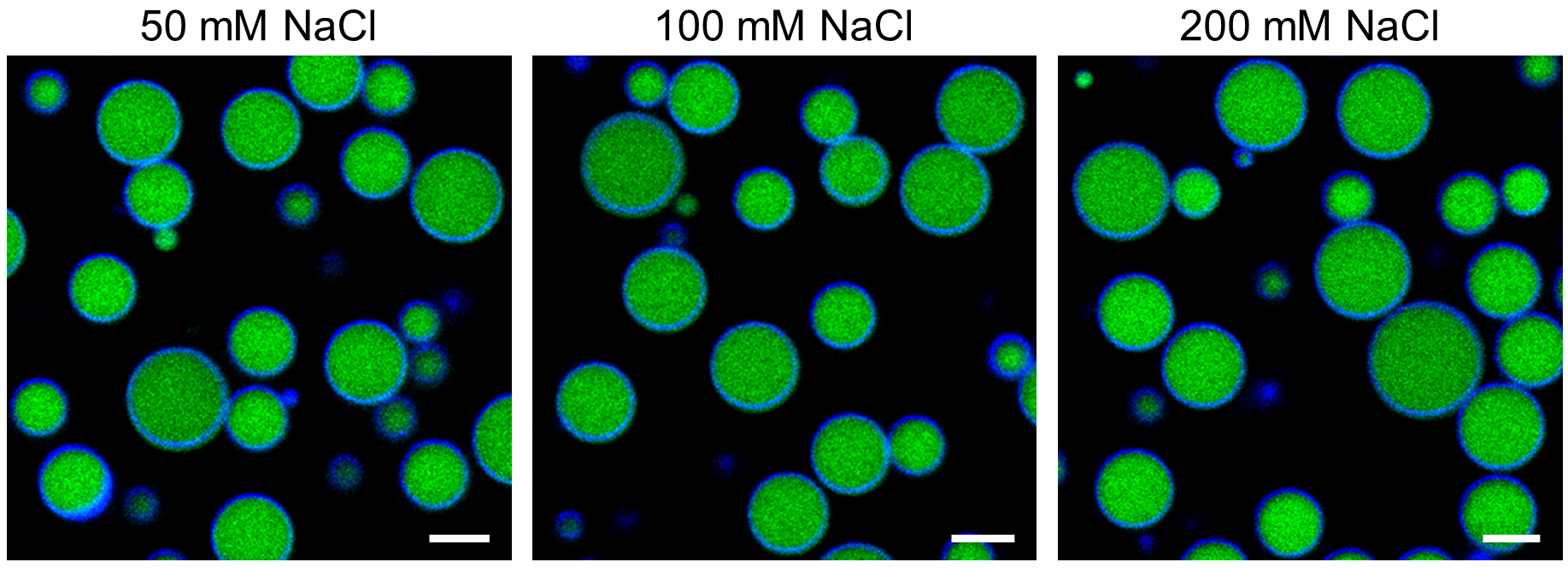


**Figure S21.** Salt resistance of generated VMCs studied by CLSM. The VMCs can be generated after addition of NaCl into NMCs at a broad concentration (final concentration of NaCl: 50 to 200 mM), indicating the high salt resistance of VMCs (green: FITC-PDDA; blue: Cy5-PAH). Scale bar: 5 µm.


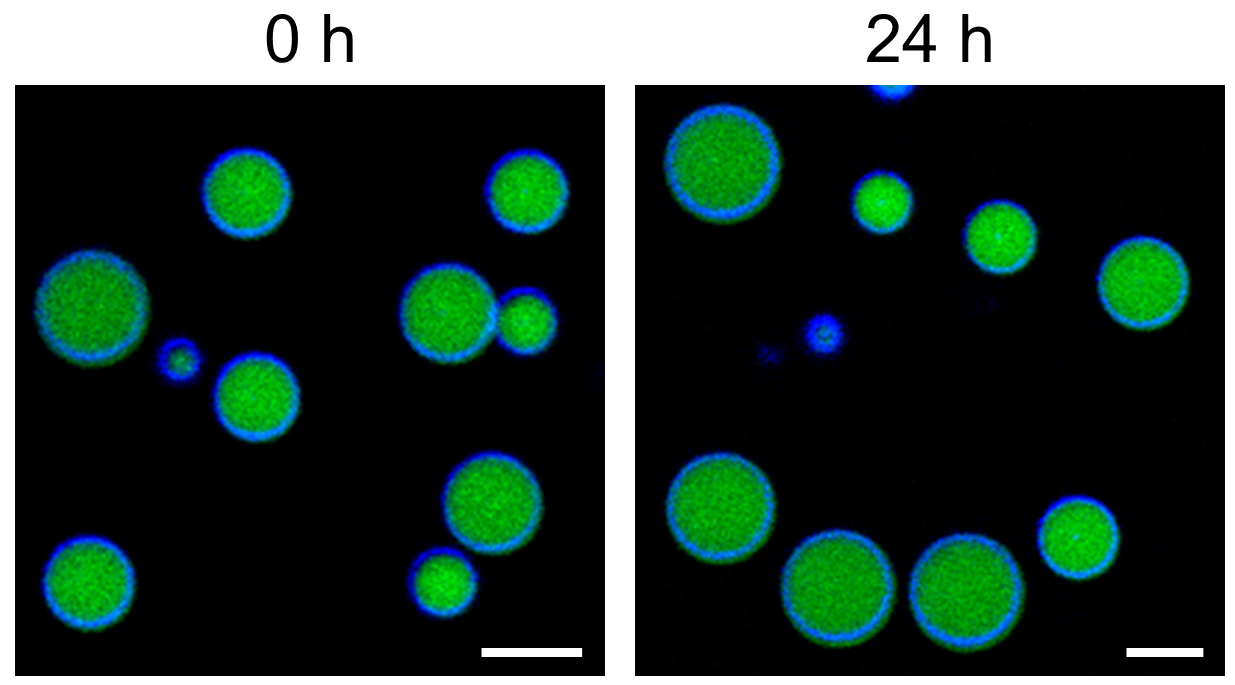


**Figure S22.** Stability of VMCs. The structural integrity of VMCs (generated at 50 mM NaCl) can be well-kept after 24 h (green: FITC-PDDA; blue: Cy5-PAH). Scale bar: 5 µm.


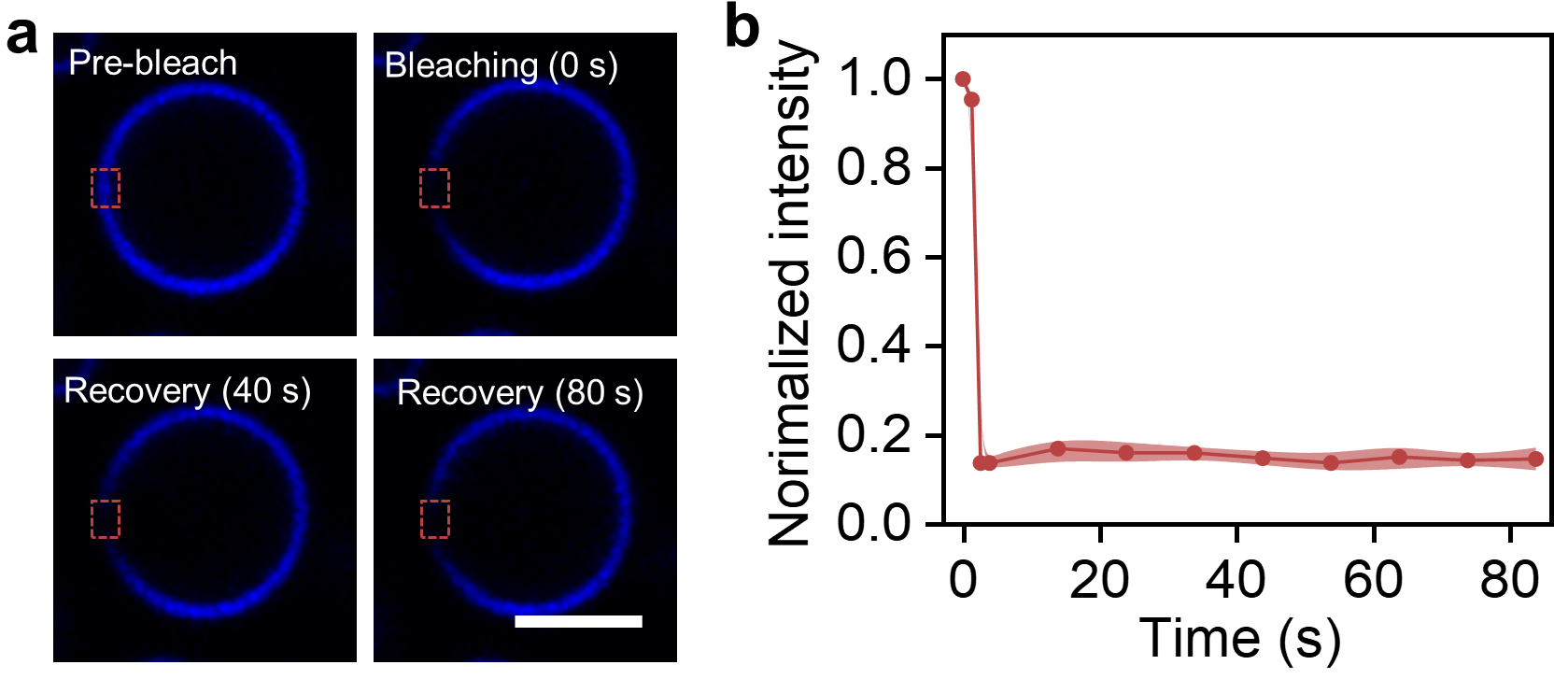


**Figure S23.** Membrane fluidity of VMCs. (a) Time series of CLSM images showing the photobleached area in the membrane of VMCs (blue: Cy5-PAH) and (b) the corresponding plot of changes in fluorescence intensity for delineated area shown in (a). The bleached area on the membrane of VMCs cannot be recovered in 80 s, indicating the illiquidity of Cy5-PAH in the PAH/ATP coacervate membrane. Scale bar: 5 μm.


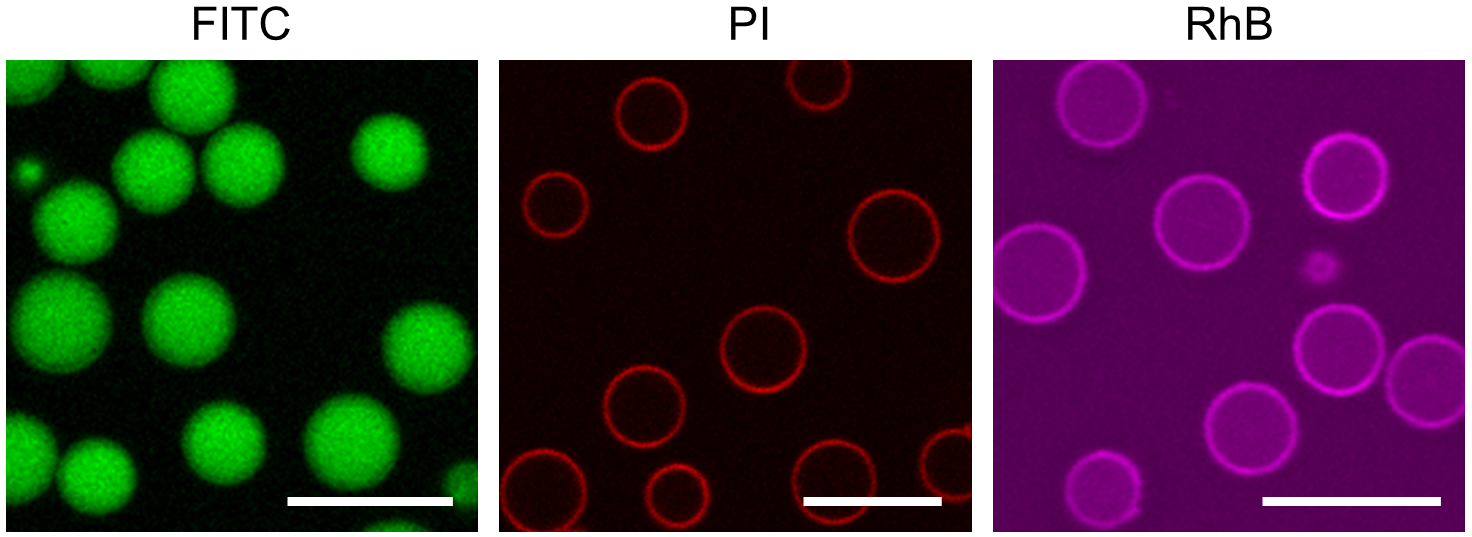


**Figure S24.** CLSM images for the sequestration behavior towards various small molecules in VMCs. As shown in the images, FITC can be primary sequestered into the lumen of VMCs, and PI is mainly located within the coacervate membrane of VMCs, RhB is sequestered into both lumen and membrane, with slightly stronger affinity toward the latter. Scale bar: 10 µm.


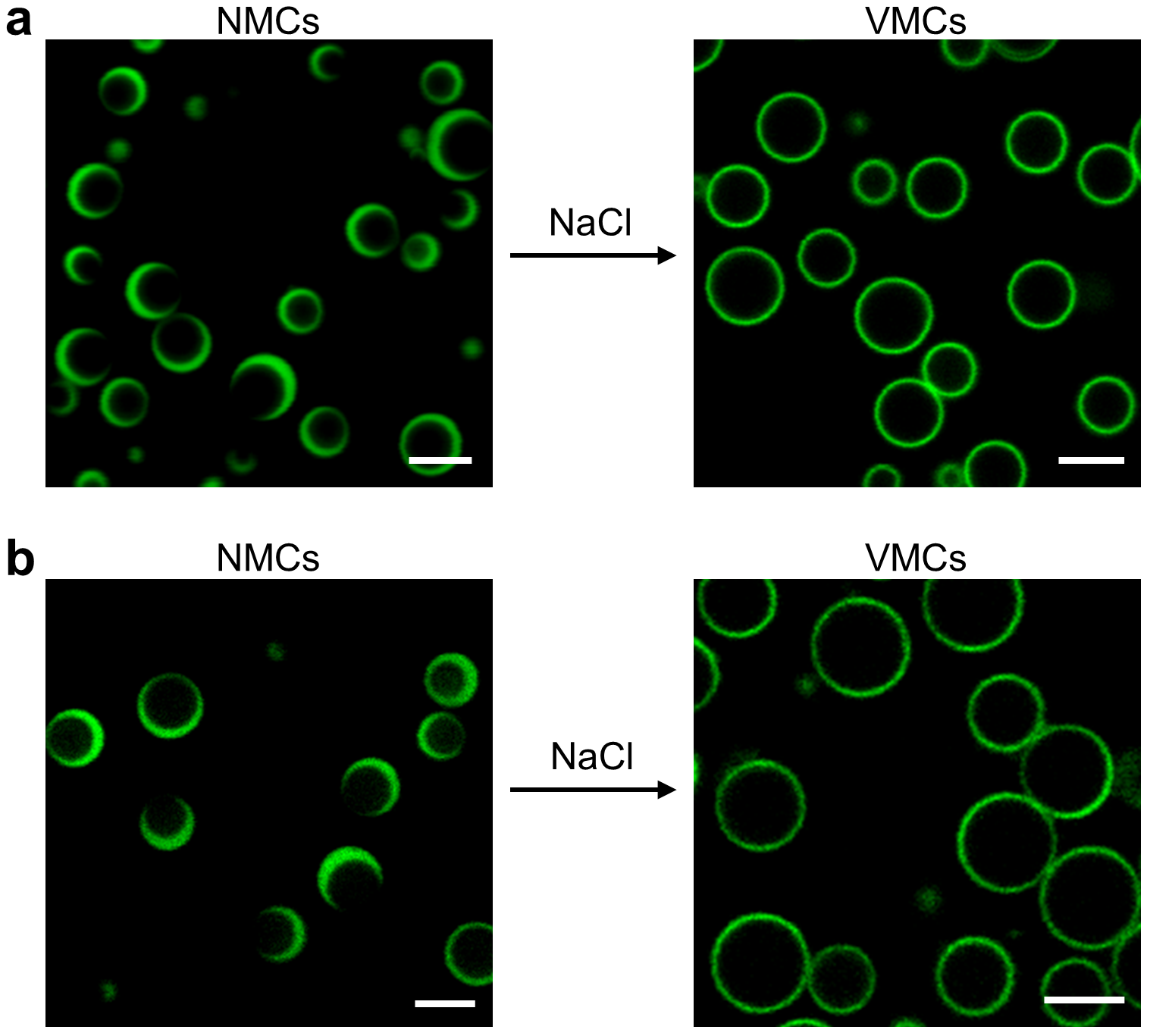


**Figure S25.** Salt-induced transition process of biomacromolecules loaded NMCs to VMCs. CLSM images showing the intra-droplet spatial transfer of (a) siRNA and (b) HRP (green: siRNA in (a) or AF 488 labeled HRP in (b)). After addition of NaCl (50 mM), both biomacromolecules are transferred from the outer PDDA/ATP phase of NMCs to the PAH/ATP coacervate membrane of VMCs, indicating the intra-droplet transfer. Scale bar: 5 µm.


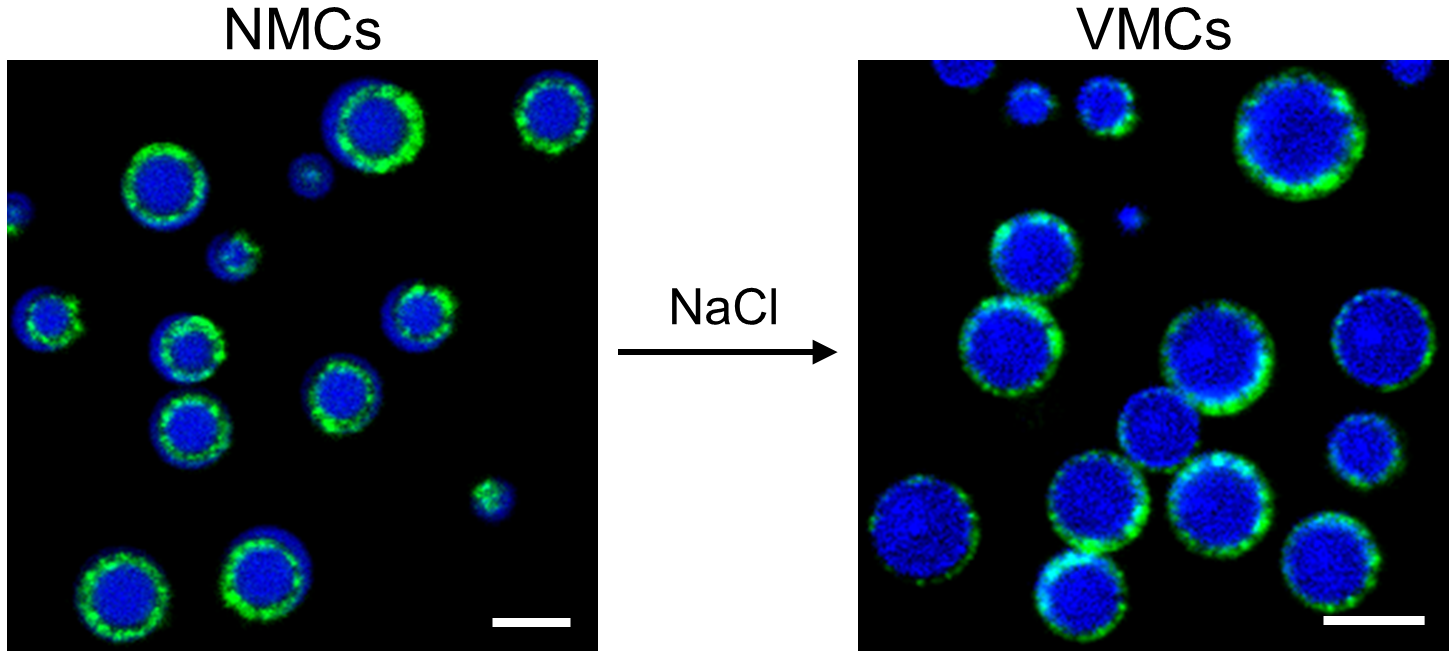


**Figure S26.** CLSM images showing the spatial transfer of beads (amine-modified polystyrene, mean particle size: 100 nm) from NMCs to VMCs. After the NaCl (50 mM)-induced transition, beads are still kept in the PAH/ATP phase (green: fluorescent from beads; blue: Cy5-PDDA). Scale bar: 5 µm.


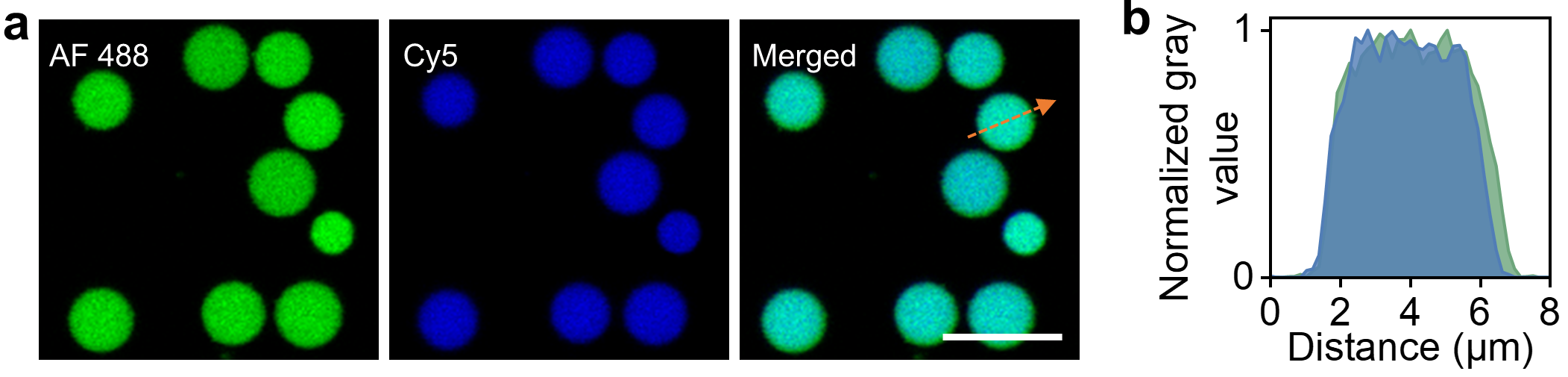


**Figure S27.** (a) Representative CLSM images after the addition of AF488 labeled esterase to MLCs (green: AF 488 labeled esterase; blue: Cy5-PDDA). (b) The normalized gray value across the dashed line in (a). As shown in this figure, the esterase can be sequestered into MLCs and show a homogeneous distribution. Scale bar: 10 µm.


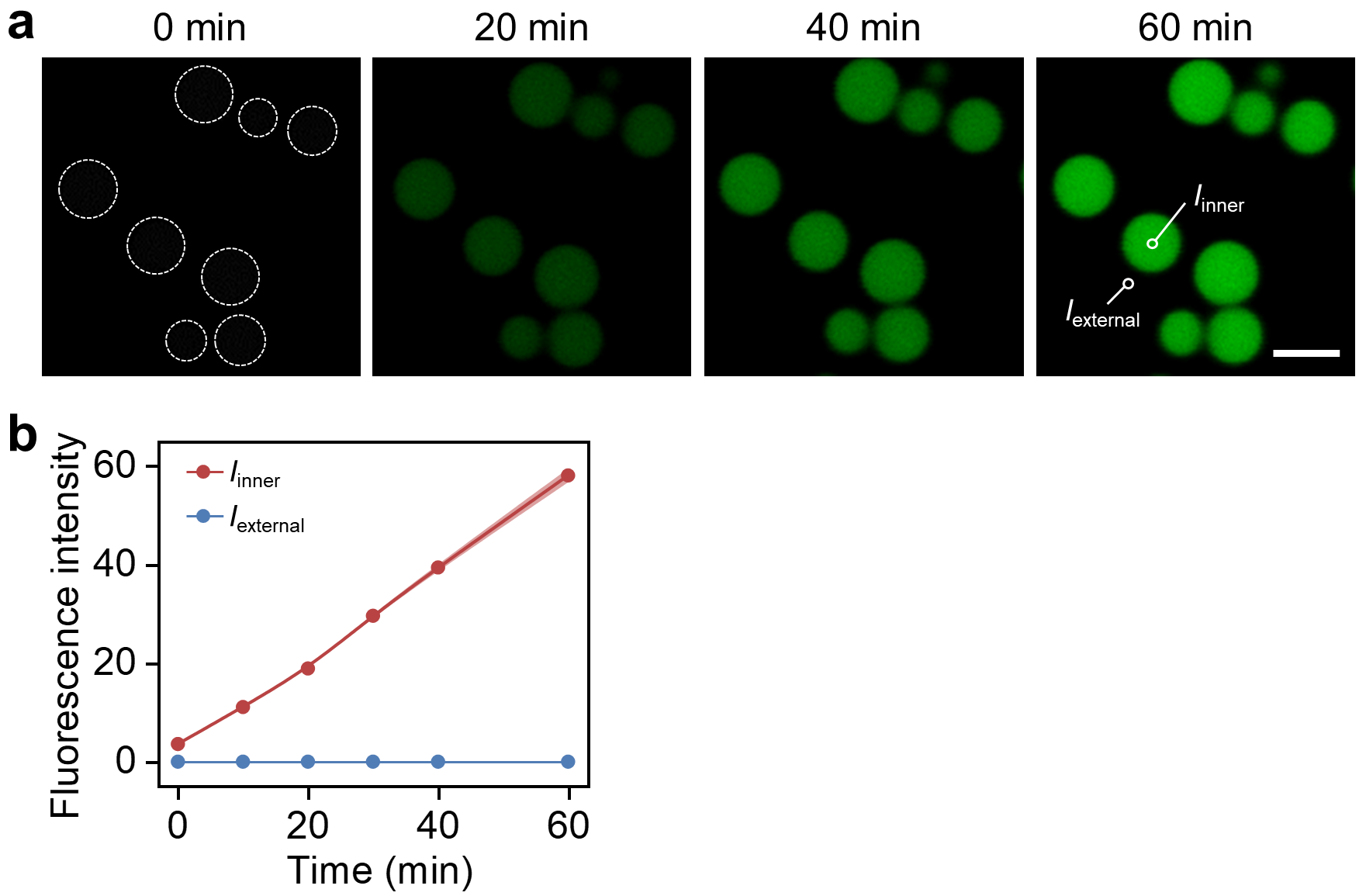


**Figure S28.** Esterase-mediated hydrolysis of CDFDA into FAM in MLCs. (a) Time series of CLSM images showing the generation of green fluorescent FAM in MLCs. (b) Time-dependent changes in fluorescence intensity for the selected areas in (a). For MLCs, after the addition of CDFDA, the homogenous green fluorescence is preferentially observed in the coacervate matrix, indicating the generation and accumulation of FAM. Scale bar: 5 µm.

**
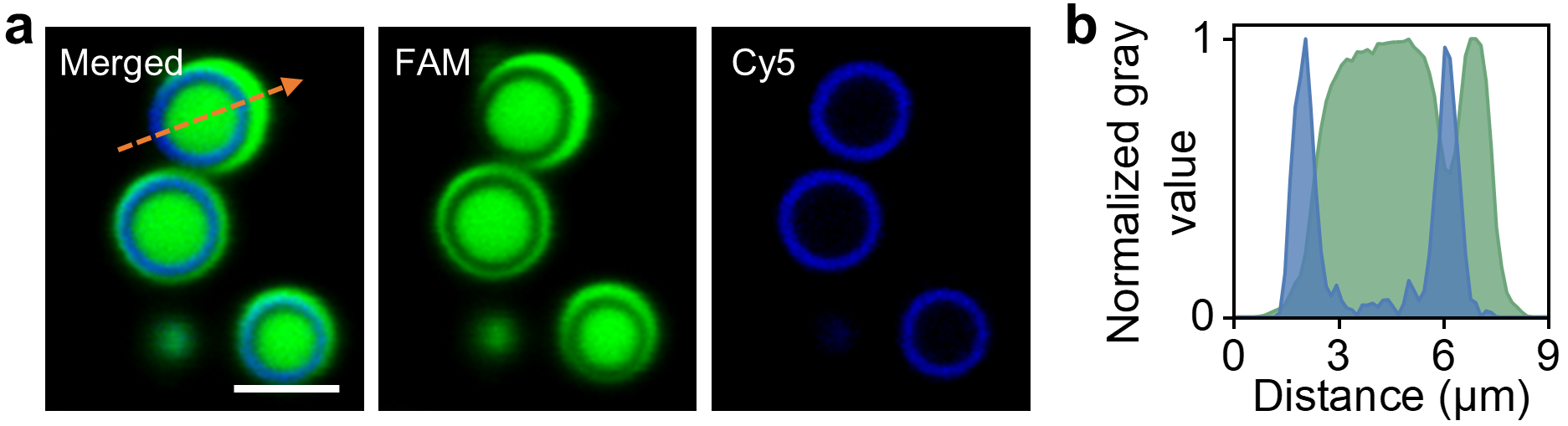
**

**Figure S29.** Analysis for esterase-mediated hydrolysis of CDFDA into FAM in NMCs as shown in **Figure 6e**. (a) CLSM images show the generated green fluorescent FAM in NMCs (green: FAM; blue: Cy5-PAH). (b) Normalized gray value across the dashed line in (a). The generated FAM is mainly sequestered in the PDDA-rich/ATP-poor phases but not enriched in the circular-like PAH-rich/ATP-rich phase. Scale bar: 5 µm.

**
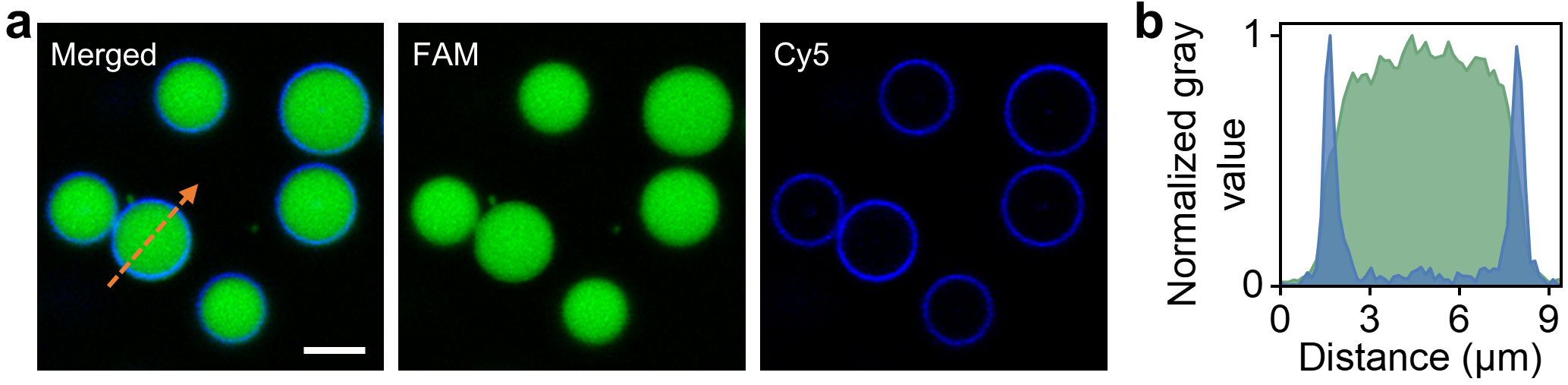
**

**Figure S30.** Analysis for esterase-mediated hydrolysis of CDFDA into FAM in VMCs as shown in **Figure 6h.** (a) CLSM images show the generated green fluorescent FAM in VMCs (green: FAM; blue: Cy5-PAH). (b) Normalized gray value across the dashed line in (a). The generated FAM is primarily sequestered in the PDDA-rich/ATP-poor lumen, and the PAH-rich/ATP-rich coacervate membrane exhibits weaker enrichment behavior towards FAM than the lumen. Scale bar: 5 µm.

**3. Reference**

(1) Zhou, Y.; Zhang, K.; Moreno, S.; Temme, A.; Voit, B.; Appelhans, D. Continuous transformation from membrane-less coacervates to membranized coacervates and giant vesicles: Toward multicompartmental protocells with complex (membrane) architectures. *Angew. Chem. Int. Ed.* **2024**, 63, e202407472.

(2) Zhou, Z.; Shen, Y.; Tang, J.; Fan, M.; Kirk, E. A. V.; Murdoch, W. J.; Radosz, M. Charge‐reversal drug conjugate for targeted cancer cell nuclear drug delivery. *Adv. Funct. Mater.* **2009**, *19*, 3580–3589.

(3) Poudyal, R. R.; Guth-Metzler, R. M.; Veenis, A. J.; Frankel, E. A.; Keating, C. D.; Bevilacqua, P. C. Template-directed RNA polymerization and enhanced ribozyme catalysis inside membraneless compartments formed by coacervates. *Nat. Commun.* **2019**, *10*, 490.
